# Impact of redeployment on healthcare staff well-being and retention: a survey of staff in the UK National Health Service

**DOI:** 10.1136/bmjopen-2025-107785

**Published:** 2026-02-02

**Authors:** Andrew Weyman, Richard Glendinning, Rachel O’Hara

**Affiliations:** 1 Psychology, University of Bath, Bath, UK; 2 Sheffield Centre for Health and Related Research, The University of Sheffield, Sheffield, UK

**Keywords:** Burnout, Professional, Psychosocial Intervention, Occupational Stress, Health Workforce, Risk management, Human resource management

## Abstract

**Abstract:**

**Background:**

The redeployment of healthcare staff from their normal place of work and duties to alternative activities is not a new phenomenon and has typically been used as a temporary measure to address capacity gaps. While redeployment supports the mobilisation of a flexible healthcare workforce, it also presents as a source of tension in relation to staff well-being and retention. This paper reports findings from a survey of staff in the UK National Health Service (NHS), exploring the impact of redeployment.

**Method:**

An online survey was administered by YouGov (2023), addressing contemporary evidence on variables impacting staff health, well-being and disposition to remain in NHS employment. The sample comprised NHS employees representing the principal healthcare job families and grades across acute hospitals, mental health, community and ambulance services. Statistical analysis (SPSS V.29.0.2.0) compared (independent samples t-test, z-test and χ^2^ test for trend) redeployed and non-redeployed staff response profiles.

**Results:**

The staff who had experienced redeployment in the 6 months prior to spring 2023 showed higher rates of submitting applications for non-NHS jobs (22%; non-redeployed staff 12%). Redeployed staff reported higher stress, lower morale and less ability to switch off from work than non-redeployed staff (p<0.01). They also showed higher ratings of symptoms of burnout (p<0.0001), higher rates of sickness presenteeism (66% redeployed; 54% non-redeployed), greater worry over current working conditions (p<0.05) and lower confidence in their improvement in the near future (p<0.01), than non-redeployed staff.

**Conclusions:**

The findings highlight the negative impacts associated with staff redeployment and challenges to staff health, well-being and disposition to remain employed in healthcare. Despite a growing consensus regarding the need to support the redeployed, evidence regarding ‘what works’ remains under-researched. Such insight is particularly pertinent given the growing interest in technological solutions for a more agile workforce, where deployment flexibility is a key feature.

**STRENGTHS AND LIMITATIONS OF THIS STUDY**

- This is believed to be the first large-scale postCOVID-19 pandemic quantitative study to explore the impact of redeployment on the health, well-being and disposition to remain as National Health Service staff.
- The sample was representative of the principal secondary care job families and was of sufficient size to support statistical testing of contrasts between redeployed and non-redeployed staff.
- Comparisons were limited to a binary comparison of redeployed and non-redeployed staff.
- While substantial, the sample could not support the exploration of potential contrasts across different segments of the secondary care workforce, for example, the type of care organisation, occupation/profession, grade, age or ethnicity, or potential confounding variables.
- The study was limited to staff employed in secondary care (acute hospitals, mental health, community care and ambulance services), and the findings may not generalise to the primary care context.

## Introduction

 ‘Redeployment’ refers to the reassignment of staff from their normal place of work and duties to alternative functions and roles. Redeployment of healthcare staff is not a new phenomenon in the UK National Health Service (NHS). Historically, it has principally been used as a short-term measure to address gaps in capacity in order to maintain minimum/sufficient staffing levels in functions experiencing staff shortages due to absence or non-normal levels of demand for care.^1^,^3^ However, there are indications that the amplified rates of redeployment necessary during the COVID-19 pandemic persist and may become an increasingly encountered feature of working life within the NHS.^4 5^

In common with other state healthcare systems, the COVID-19 pandemic led to unprecedented rates of redeployment within the NHS, with widespread reassignment of healthcare workers to often unfamiliar work environments and roles. PreCOVID-19, despite redeployment being an established feature, particularly within secondary care delivery, the issue of impacts on staff well-being, performance, retention and care quality appears to have received relatively little institutional or research attention with notable exceptions (eg, Donnelly^1^; Saville *et al*^2^). COVID-19 working conditions gave rise to notable redress in this respect, within the UK and internationally, typically as a component of broader perspectives on staff experiences, with a strong focus on the intuitively most challenging redeployments to emergency, intensive and end-of-life care.^36^,^9^

Studies report an array of negative impacts on redeployed personnel arising, directly or indirectly, from pandemic working conditions and arrangements. While positive staff experiences are also reported (eg, opportunity to enhance skills and experience),^7 9^ negative impacts dominate the study findings. Despite variability in study population (profession; type of provider organisation), sample size and the extent of inclusion of key salient comparators (notably redeployed vs non-redeployed staff, preCOVID-19 vs postCOVID-19, voluntary vs involuntary assignment), consistent headline findings include degraded job satisfaction,^10^ amplified rates of stress and symptoms of burnout,^11^,^16^ feelings of detachment/isolation and poorer relations with managers,^10^ feeling undervalued,^7^ impacts on intention to quit^7 9 17^ and reduced patient satisfaction.^18^ The majority of studies focus on single health professions or functions during the height of the COVID-19 pandemic. They characterise staff redeployed during that period as a more vulnerable ‘…high-risk group, so should be a target for health and well-being interventions’ (p. 378).^16^

The profile of demand for secondary care and prevailing conditions during the pandemic is reported to have sponsored a more centralised command and control structure, particularly within secondary care, with senior managers taking a more strategic role in staff deployment decisions. An associated feature was increased employer adoption of e-rostering technology, reported to have ‘…proven instrumental…during the COVID-19 response to redeploy staff quickly to support areas in need.’^19^ However, the postpandemic persistence of this approach to staff deployment has been characterised as a cultural shift from historical norms that conflicts with staff expectations and devolved department-level decision-making (eg, by nurse managers).^3^

Hartley *et al*^3^ concluded that while redeployment is key to mobilising a flexible workforce within healthcare, it presents as being in some tension with the need to stabilise/enhance rates of staff retention. Notably, these authors report increased pushback among staff redeployed involuntarily as the pandemic matured, on grounds of unfairness and fundamental unattractiveness, leading to stress for line managers tasked with balancing broader service demands with the needs and preferences of their team.

This paper reports findings from a survey of NHS staff in spring 2023. It is supported by reference to three earlier waves of the survey in winter 2020 (wave 1), spring 2021 (wave 2) and spring 2022 (wave 3). The issue of staff redeployment was a component of a broader exploration of postpandemic employee health, well-being, attachment and disposition to remain in NHS employment, from the perspective of staff retention. As such, the research did not set out to establish or test a theoretically informed basis for the impact of redeployment; rather, the contrast between redeployed and non-redeployed staff emerged as a noteworthy feature of our study of variables associated with staff disposition to remain in NHS employment.

A point of contrast with the body of pandemic-initiated redeployment research into impacts on staff health and well-being is that the data gathered in our wave 4 survey covers the return to routine care delivery in 2023, which is the focus of this paper.

## Aims

At the point of commissioning (autumn 2020), the research set out to provide human resource strategy and policy-relevant insight into:

- The impact of the COVID-19 experiences on employees’ strength of attachment, commitment and capacity to remain in NHS employment.
- The relative salience and strength of *push* and *pull* variables on staff stay versus leave intentions and behaviour.
- What might need to change to motivate/enable current employees to remain in NHS employment.
- The need, nature and scope for intervention to maintain/enhance retention rates, and how this might vary across different employee demographics.

However, the scope of data gathering broadened over the course of the study (from winter 2020 to summer 2023) from its initial focus on primary impacts arising from COVID-19 in 2020/2021 and its legacy to include persistent and emergent features of the postpandemic work environment, including staff shortages, workload, job demands, working conditions, pay and other background climate factors with the potential to impact staff health, well-being, resilience, capacity and disposition to remain in NHS employment.

Findings from our initial headline analysis of survey data, reported elsewhere,^20^ indicated that redeployed staff exhibited a consistently more negative response profile across a high proportion of the variables explored in the survey. This provided the justification for the deeper exploration of the data reported in this paper.

## Method

Epistemologically, the content of the survey reflected a systems perspective, focused on contextual influences on employee health, well-being and disposition to stay or leave. Variables explored encompassed structural elements (eg, working arrangements and practices, workload and working hours), workplace climate and cultural as well as social normative variables, extending to more impressionistic psychosocial variables (eg, staff perceptions of feeling valued by key stakeholders).

Taking a risk mitigation and control perspective, the focus was on identifying associative influences that can be characterised as incubating precursors,^21^ with the potential to challenge staff resilience to prevailing working conditions/arrangements and diminish their attachment to NHS employment. The focus for leaving was on exits from NHS employment, rather than internal (within NHS) transitions (ie, the potential net loss to NHS capacity). The core objective was to identify priority issues for intervention, nuanced by identifying the demographics and functional groups of employees that exhibited amplified vulnerability,^22^ with redeployed staff constituting one of an array of segments.

### Patient and public involvement

Patients and/or the public were not involved in the design, conduct, reporting or dissemination plans of this research.

## Materials

Topics explored within the survey (table 1) were grounded in published research evidence on variables identified as impacting staff health, well-being and disposition to remain in NHS employment,^23^ ^20 24^ supplemented with input from health sector stakeholders, including the Department of Health and Social Care, NHS England, the Royal College of Nursing, the Royal College of Midwives and UNISON. In view of the relatively large number of variables that needed to be addressed within the survey of staff retention and reflecting the general approach within the annual NHS staff survey, questions were configured as discrete stand-alone items, rather than a compendium of established psychometric scales. This was for three reasons: (1) the use of generic scales risked obscuring NHS context-specific elements, most pertinently variables arising from the response to the COVID-19 pandemic; (2) the implications for completion time would have significantly restricted the range of variables that could be addressed and (3) to maximise the funder’s human resource policy-relevant insight, the question sets at each wave needed to be sufficiently agile to take account of emergent and recessive issues from the height of the pandemic in 2020 to the return to routine care provision by late 2022/early 2003.

**Table 1:** Survey themes and topics explored—survey wave 4 (spring 2023)

Themes	Topics—psychosocial	Topics— structural
Reasons why staff stay	Job (dis)satisfaction	Workload
Reasons why staff leave	Support	Resources and staffing levels
	Employer	
	Managers	
What has got better/worse	Physical health	Working hours
Worries and concerns	Mental health	Flexible working
Confidence in the future	Morale	Redeployment
Future work/retirement aspirations	Burnout	Pay and financial well-being
Non-NHS job-seeking behaviour	Sickness presenteeism	Career and promotion opportunities
Strength of attachment to the NHS	Work-home life balance	Cost of living
What has changed and what needs to change	Recognition of effort/contribution	
	Feeling undervalued	
	Government	
	Senior managers	
	Line manager	
Aggression from patients/public		

NHS, National Health Service.

To reflect evolving working conditions, policy changes, fluctuations in the profile and nature of demand for care and arising funder interests (eg, proposed mandatory vaccination of staff, availability of personal protective equipment and changes to reference timeframes), approximately 5%–10% of the question set was subject to detailed revision at each wave.

The presentation order within questions was randomised to the extent possible within their respective logic structures (all grid questions were randomised) and consistent with market research sector norms for large-scale public policy and commercial surveys.

A copy of the wave 4 survey question set is provided in online supplemental appendix 4. This includes a supplementary note on detailed changes to question wording and inclusion at each wave.

### The sample

The survey was administered by YouGov. Participants were YouGov panel members who were directly employed by the NHS at the time of the survey. YouGov panel members receive a points-based accrual for each survey they complete. A typical accrual for participation in multiple surveys on multiple issues is approximately £50 per annum.

The sample (table 2) comprised the principal healthcare job families (mainly health professionals—medical/dental, allied health, nursing and paramedics, as well as care support, scientific and technical, administrative and estates/ancillary) and grades directly employed in secondary care services (acute hospitals, mental health, community and ambulance).

**Table 2:** Obtained sample breakdown by occupation (%) (waves 1–4)

	Wave 1 (n=1962)	Wave 2 (n=2240)	Wave 3 (n=1538)	Wave 4 (n=1653)
Nursing/nursing support/midwives	30	30	30	31
Allied health	18	15	15	14
Medical and dental	12	9	10	9
Scientific and technical	7	6	6	6
Ambulance	3	3	3	3
Clinical management	1	1	1	1
Commissioning managers	>0.5	1	1	1
Ancillary and support	2	2	2	2
Administration, technical and corporate services	27	29	28	29
Other	>0.5	3	3	3

The sample was stratified by occupational group and weighted by age, gender identity, ethnicity and region, with strong representation by occupational group and type of care provider organisation. The samples for waves 3 and 4 were England only, whereas waves 1 and 2 were UK-wide. Preanalysis checks revealed no differences (proportions, means and SD) in the response profiles between England and the devolved nations, indicating that the respective data sets could be treated as homogenous.

### Analysis

The analysis focuses on the fourth wave of data capture (spring 2023) due to its greater contemporary relevance to working conditions and arrangements following the postCOVID-19 return to more normal care delivery from late 2022/2023. At wave 4, exposure to redeployment was determined on the basis of the response to the question ‘In the last 6 months, how often, if at all, have you been redeployed to a different location, department or team?’. The reference period at waves 1–3 was ‘since March 2020’. This change was introduced due to the increasingly distal anchor of the beginning of the pandemic and the transition to more normal working conditions and arrangements.

The relatively large sample allowed statistical testing of redeployed and non-redeployed staff contrasts with respect to an array of variables that have also been reported in pre2020 and post2020 small-sample qualitative redeployment studies.^825^,^27^ It is also believed to be the first to compare substantial samples of staff with experience of recent redeployment following the return to more normal working conditions postCOVID-19.

In addition to descriptive statistics, inferential statistics tests (independent samples t-test, z-test and χ^2^ test for trend) using SPSS V.29.0.2.0^28^ were applied to compare redeployed with non-redeployed staff. Contrasts are explored for rates of applications for non-NHS jobs and sickness presenteeism; ratings of stress, morale, ability to switch off from work and symptoms of burnout and worry over current working conditions and confidence in improvement to working conditions.

Cross-wave comparisons are limited to the contemporary relevant issues of whether rates of redeployment (voluntary and involuntary) have reduced since the height of the pandemic in 2020/2021; the proportion of staff (redeployed and non-redeployed) who would recommend working for the NHS and ratings of symptoms of burnout (added to the survey at waves 3 and 4).

## Results

At wave 4, 24% of respondents said that they had been redeployed at some point in the previous 6 months. Of these, 25% reported this happening often, and 75% occasionally. 30% of redeployments were reported to have been voluntary and 70% involuntary, with an indication of a rising rate of involuntary redeployments across the four waves. Statistical testing (Chi2 Test for trend) confirmed a rising profile for rates of involuntary redeployment across the four waves of the survey (X 2 (1, N = 794) = 15.45; p =0.0001).

### Exploration of alternative (non-NHS) employment opportunities

**Figure 1:**
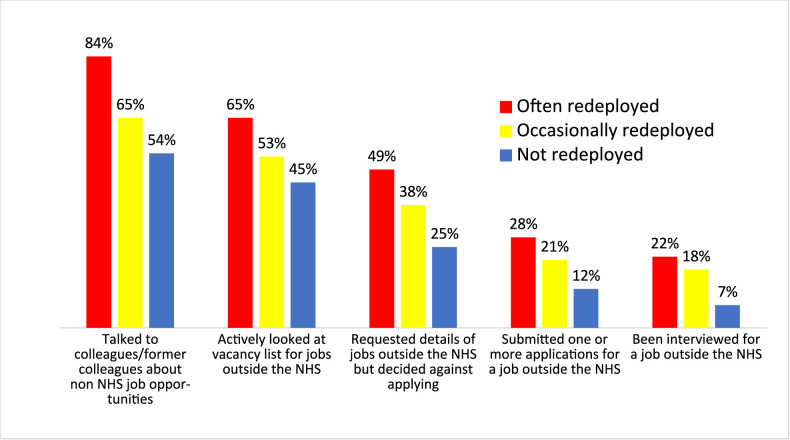
Proportion of staff (often, occasionally or never recently redeployed) engaging in exit precursor behaviour(s) in the previous 6 months (wave 4. NHS, National Health Service.

Arguably, the most salient question relating to the impacts of redeployment, from the perspective of future NHS capacity, relates to the linkages to quit rates, particularly exits to non-NHS employment. Based on the assumption that exit decisions are potentially underpinned by prior contemplation and preparatory precursor behaviours, respondents were asked, ‘*What steps (if any) have you taken towards non-NHS employment in the last 6 months?’* referenced to a 6-point behavioural ladder (Guttman-type) scale, with anchors that ranged from ‘*talked to colleagues/former colleagues about job opportunities outside the NHS*’ to ‘*been interviewed for jobs outside the NHS*’ (figure 1).

The data depicted in figure 1 show higher rates of engagement in exit-precursor behaviours among staff who reported having been redeployed compared with those who had not. The ‘often’ group exhibited higher rates than the ‘occasionally’ redeployed, suggesting a redeployment exposure effect. However, the modest size of the often-redeployed sample (n=89, at wave 4) renders this finding tentative. In recognition of this, it was considered prudent to limit deeper exploration to dichotomous comparisons between the redeployed (frequently and occasionally) and the non-redeployed.

#### Non-NHS job application rates

At wave 4, the redeployed staff rate of submitting (one or more) non-NHS job applications (22%) was almost double the non-redeployed rate (12%). Among nurses, the most frequently redeployed segment, relative differences in application rates showed alignment with the all-staff profile (redeployed 17%; non-redeployed 10%).

#### Job satisfaction, morale and mental health

At wave 4, referenced to the 6-month period prior to spring 2023, a similar dichotomous comparison revealed a negative rise in ratings of stress among the redeployed and lower ratings of *morale*, *ability to switch off at home and the extent to which I enjoy my job* (5-point scale: *a lot better* to *a lot worse,* during the previous 6 months) (online supplemental appendix 1).

**Figure 2:**
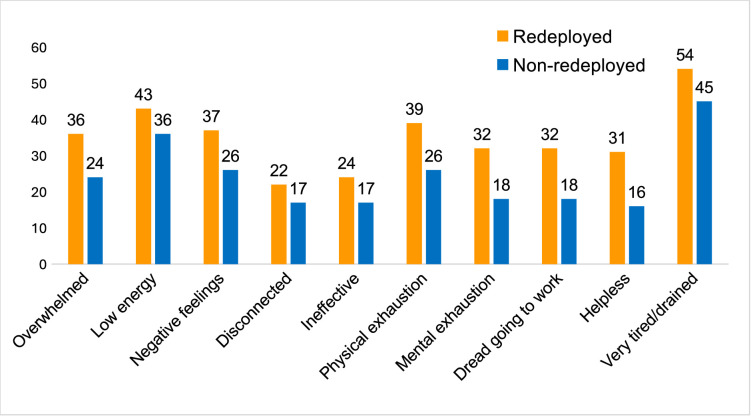
Wave 4—percentage of redeployed and non-redeployed staff reporting burnout by symptom.

At waves 3 and 4, participants were asked if they experienced any of an array of widely cited indicators of burnout (feeling overwhelmed, drained, helpless, experiencing low energy, negative feelings, feeling disconnected, physical exhaustion, mental exhaustion, dreading going to work and (added at wave 4) loss of empathy with patients) *most or every day* during the previous 6 months. This revealed rises from wave 3 to 4 and consistently higher rates among the redeployed, with greater contrast at wave 4. The mean difference was 11.5 percentage points, with a contrast of more than 10 percentage points for 7 of the 11 variables (figure 2).

At wave 3, the mean difference was 5.6 percentage points, with none greater than 10 percentage points. Formal testing (independent samples t-tests) confirmed a difference (*p*<0.05) between redeployed and non-redeployed for each variable at waves 3 and 4, except for mental exhaustion and feeling helpless at wave 3. At wave 4, *t*(1652) ranged from 2.97 to 6.93, p=0.0001; at wave 3, *t*(1561) ranged from 2.03 to 3.42, p=0.004–0.0006 (online supplemental appendix 2).

#### Sources of worry and concern

Redeployed staff also exhibit higher mean ratings of worry (10-point, low-to-high scale) and lower confidence (4-point, low-to-high scale) in the future over an array of variables relating to their health and well-being and the future of the NHS.

**Figure 3:**
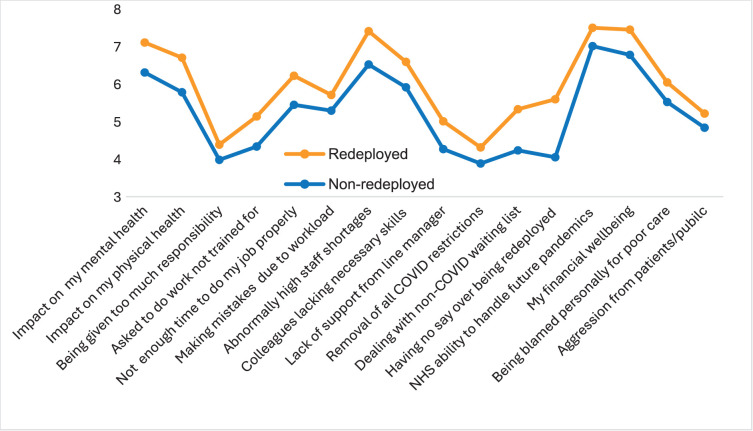
Redeployed versus non-redeployed mean ratings of worry—wave 4. NHS, National Health Service.

The profiles of ratings for worry (not at all: 1, extremely: 10) showed high alignment between the redeployed and non-deployed (ie, at the level of rank order). However, the magnitude of worry was greater among the former across all variables explored (figure 3). It is worth noting that the most marked contrast related to worry over future redeployment, *‘Not having any say about being redeployed to a different role or team*’, which showed a large effect size (Cohen’s *d*=4.78). Formal testing (independent samples t-test) revealed significant differences <0.05 across all variables in the set except ‘*Aggression from patients and the public’* (online supplemental appendix 3).

#### Confidence in improvements to working conditions

**Figure 4:**
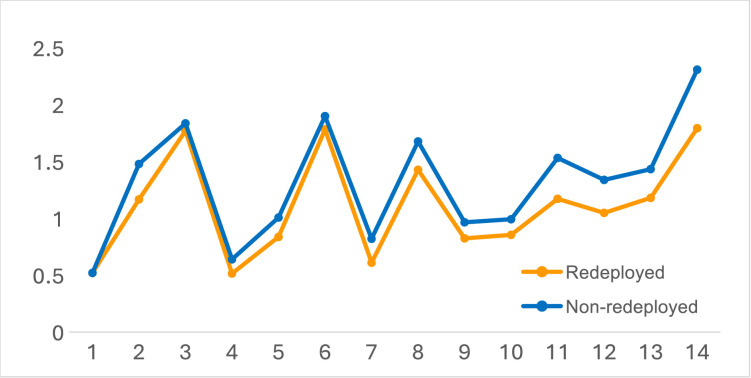
Redeployed versus non-redeployed confidence in working conditions and impacts over the next 12 months (wave 4).

Respondents were asked to rate their confidence in positive change *‘over the next 12 months’* to working conditions, arrangements, institutional capacity to meet the demand for care and impacts on their quality of working life (referenced to a 5-point, low-to-high scale; see figure 4 and table 3).

**Table 3:** Redeployed and non-redeployed contrasts in mean ratings of confidence in working conditions and arrangements in the next 12 months (ie, summer 2024)

Item	Scale 1–4 (high to low)	Redeployed		Non-redeployed		df=1653		
	t-test independent measures	M	SD	M	SD	*t*	P value	Cohen’s *d*
14	I will be working with people that I know on my next shift	2.21	0.91	1.69	0.79	10.79	<0.001	0.63
11	I will be satisfied with the standard of care I am able to deliver**	2.76	0.80	2.47	0.84	7.45	<0.001	0.43
2	Your organisation will proactively support your health and well-being	2.83	0.81	2.52	0.83	6.54	<0.001	0.38
12	Staff in your team will stay working in the NHS	2.95	0.80	2.66	0.82	6.11	<0.001	0.35
13	The NHS organisation I work for will be able to deliver an acceptable standard of patient care	2.82	0.82	2.49	0.83	5.26	<0.001	0.30
8	My own future working in the NHS	2.57	0.91	2.32	0.85	4.98	<0.001	0.29
7	Staffing levels in my trust/NHS organisation will improve	3.39	0.72	3.18	0.75	4.97	<0.001	0.28
5	My stress levels will go down	3.16	0.72	2.99	0.76	3.91	<0.001	0.23
9	The NHS will be able to cope with the demand for non-COVID-19 healthcare	3.18	0.71	3.03	0.77	3.24	<0.001	0.19
4	My daily workload will go down	3.49	0.65	3.36	0.69	3.17	<0.001	0.18
10	NHS resources will be prepared for a further wave of the COVID-19 pandemic	3.14	0.79	3.01	0.83	2.98	<0.001	0.17
8	We have seen the worst of the COVID-19 pandemic	2.22	0.78	2.09	0.77	2.59	0.009	0.15
3	The vaccine programme will be effective in controlling COVID-19	2.23	0.77	2.16	0.83	1.47	0.137	–
1	The NHS will get the funding resource it needs*	3.48	0.74	3.48	0.72	0.01	0.994	–

M= Mean; t= t-statistic

*df=1603; **df=1340

NHS, National Health Service.

Reflecting the profile for other variables, at the level of rank order, the redeployed and non-redeployed exhibited an almost identical profile, but the redeployed were consistently more negative in absolute terms for 12 of the 14 variables explored (ie, the redeployed were less confident of improvement).

#### Sickness presenteeism

At wave 4, the redeployed recorded higher ratings of pressure from their employer to work extra hours over the previous 6 months (4-point scale: ‘*not at* all’ to ‘*a lot*’). 20% of the redeployed reported ‘*a lot*’ of pressure, compared with 7% for the non-redeployed.

Reports of instances of sickness presenteeism during the previous 6 months, while high in both groups, also showed a contrast: 66% redeployed and 54% non-redeployed. Further examination of respondents’ rationale for working while sick showed contrasts for seven of the nine variables explored (4-point scale ‘not at all’ to ‘a lot’; table 4), with medium effect sizes for exceeding the sickness-absence allowance, loss of pay and the manager’s reaction to absence.

**Table 4:** Redeployed and non-redeployed contrasts in mean ratings of motivators of sickness presenteeism

Sickness presenteeism wave 4 (scale 1–5; low to high)	Redeployed (n=246)		Non-redeployed (n=673)		df=917		
t-test independent measures	Mean	SD	Mean	SD	*t*	P value	Cohen’s *d*
Exceeding my sickness absence days allowance	2.10	0.87	2.38	0.77	4.22	<0.001	0.34
Losing pay	2.45	0.80	2.71	0.75	4.12	<0.001	0.34
My manager’s reaction	2.15	0.80	2.40	0.82	4.11	<0.001	0.31
Letting patients/service users down	1.74	0.72	1.90	0.80	2.68	0.007	0.20
Falling behind with my work	1.53	0.66	1.92	0.72	2.49	0.013	0.19
Impact my attendance record	1.87	0.83	2.02	0.86	2.33	0.020	0.17
Getting a bad reputation	2.18	0.80	2.32	0.86	2.13	0.032	0.16
No one else could cover my role	1.84	0.82	1.82	0.83	0.55	0.582	–
Extra burden placed on colleagues	1.53	0.66	1.60	0.79	0.17	0.174	–

t= t-statisitc.

#### Recommend working for the NHS

**Figure 5:**
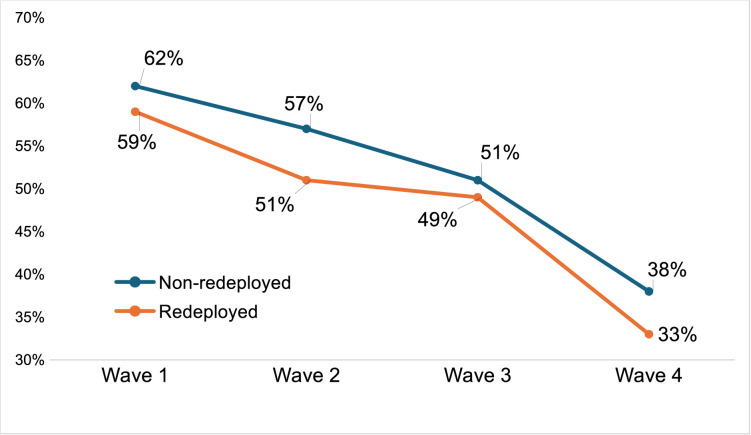
Redeployed versus non-redeployed ‘I would recommend working for the NHS to others’ (wave 4).

The proportion of respondents who agreed with the statement ‘I would recommend working for the NHS to others’ (5-point scale, from strongly agree to strongly disagree) showed a marked negative trend across the four waves, dropping from approximately 3:5 (wave 1) to 1:3 (wave 4), with a 14 percentage point drop over approximately 12 months between waves 3 and 4. The proportion of redeployed staff agreeing with this statement was lower than that of the non-redeployed at each wave (figure 5).

Formal testing (t-test independent measures; see table 5) revealed differences in the agree proportions p<0.05.

**Table 5:** Redeployed and non-redeployed contrasts in mean ratings of recommending working for the NHS to others

Recommend working for the NHS to others Wave 4 (scale 1–5; strongly agree to strongly disagree)								
t-test independent measures*								
Redeployed	Wave 1		Wave 2		Wave 3		Wave 4	
	Yes	No	Yes	No	Yes	No	Yes	No
Mean	2.48	2.35	2.66	2.47	2.69	2.57	3.08	2.90
SD	1.17	0.92	1.16	1.06	1.09	1.09	1.20	1.09
N	423	1488	544	1675	350	1187	384	1249
*t*	2.44		3.75		1.67		2.90	
P value	0.004		0.00002		0.010		0.004	

* Discrete t-tests were performed due to between-wave differences in response reference timeframe (since March 2020 for waves 1–3; last 6 months for wave 4).

## Discussion

The survey covers the period after the emergence of COVID-19, so it cannot directly answer the question of whether current rates of redeployment are higher than before the pandemic. But, given that it is widely accepted that the pandemic led to unprecedented rates of redeployment,^3 7 8^ our finding that rates have not reduced between 2020 and 2023 would seem to lend weight to the claim of a postpandemic rise. Notwithstanding this, our focus was not on determining whether rates had risen or not but on exploring the claims from predominantly qualitative research of an array of negative impacts on staff health, well-being and commitment to remain in NHS employment arising from the experience of redeployment. This has relevance to NHS staff retention and care provider capacity, irrespective of the rate of redeployment, but potentially assumes greater salience where the rate is high or set to rise.

Our findings provide a strong indication that comparisons between redeployed and non-redeployed staff reveal significant contrasts across an array of widely cited influences on staff well-being and disposition to remain within the NHS. At wave 4, the redeployed showed higher rates of exploring non-NHS employment opportunities, higher rates of submitting job applications for employment outside the NHS (22%), almost double the rate of the non-redeployed (12%), and were less likely to recommend working for the NHS (33% redeployed; 38% non-redeployed). With respect to the latter, both groups showed a marked drop in the proportion of positive responses between waves 3 and 4. Notably, consistent contrasts include a more negative profile with respect to symptoms of burnout and confidence in improvement to working conditions in the near future. In sum, it appears that features of the experience and status of redeployment can amplify established challenges to staff well-being and commitment to remain. The relative salience of these variables for the redeployed and non-redeployed presents as common and consistent at the level of rank order but is negatively amplified for the redeployed. Some notable instances of divergence include worry over being deployed ‘*to work I have not been trained for*’, ‘*feeling overwhelmed*’*,* ‘*physical exhaustion*’, ‘*mental exhaustion*’ and ‘*satisfaction with the standard of care I am able to provide*’*.*

The presence of an association between staff reports of symptoms of burnout and redeployment mirrors the findings from several studies on the impacts of COVID-19. Similarly, the indication of negative impacts on the redeployed arising from staff shortages, lack of familiarity with the work environment and staff, amplified rates of physical and mental exhaustion, anxiety over skill set and lower morale.^7 10 12 15^ However, no references were found in the literature regarding our detected association between sickness presenteeism and redeployment, which might have been expected as a concomitant of reports of increased sickness absence.^3^ The exploration of the underpinning rationale for sickness presenteeism indicated the primacy of three variables: ‘exceeding my sick-day allowance’, ‘loss of pay’ and ‘the manager’s reaction’. Of these, it is possible that the first two are linked. It is also the case that staff suffering persistent ill-health and/or more frequent exposure to causes of ill-health have greater occasions to work while sick, simply because they have a higher proportion of days within the reference period when they are unwell. The third, however, points to the relationship with their line manager. Published findings variously point to a lack of support for the redeployed from line managers, lack of clarity over who their line manager is and degraded communication with line managers.^8 29 30^ Our findings showed a difference with respect to worry over the relationship with the line manager, although the effect size was modest.

A recognised feature of the experience and/or spectre of redeployment is its association with uncertainty.^9^ Staff routinely have little certainty or control in relation to whether, when and where they will be deployed. The linkage between uncertainty giving rise to employee worry is well established^30^ and has previously been identified as a source of healthcare staff concern and stress contributing to burnout^29^ with negative impacts on morale.^7^ Both appear to be reflected in our findings, with the redeployed reporting lower levels of morale and greater worry than non-redeployed staff on all 15 of the variables explored. Mirroring this, their ratings of confidence in improvements to working conditions and associated impacts showed a similar contrast for 13 of the 15 variables examined.

The findings on worry and confidence in the future appear to reflect a generalised degradation of experience. Given that it is not immediately apparent why these response profiles might arise as a direct consequence of redeployment, there are a number of standout variables that reflect alignment with established insights and/or intuitive associations, where contrasts show medium and high effect sizes: ‘*not having enough time to do my job properly*’; ‘*satisfaction with the standard of care I am able to give*’^10^; ‘*being given too much responsibility*’; ‘*being asked to do work I have not been trained for*’^7 15^; ‘*aggression from patients*’; ‘*blame for poor care*’ and ‘*I will be working with people I know on my next shift*’.^9^ Speculatively, reflecting the conclusions of others,^7 8 31^ and given our finding that 70% of deployments were reported as involuntary, it is perhaps plausible that the uncertainty individuals experience over *if, when* and to *where* they will be redeployed contributes to a generalised orientation of amplified angst and despondency.

## Human resource policy implications

Rising rates of demand for care in the presence of persistent staff shortages and strong drives for efficiency mean that amplified rates of redeployment seem likely to persist and become a new normal. Our findings, and those of others, highlight an associated array of negative impacts on staff health, well-being and disposition to stay in the NHS for redeployed staff. Thus, the redeployed present as a higher-risk cohort within an already vulnerable workforce. If left unchecked, an arising inference is that redeployment, as a necessary institutional coping measure for managing staff shortage, may exacerbate these negative impacts on staff, with the potential to lead to underperformance, higher rates of sickness absence and early exit from the NHS. In other words, measures to manage staff shortages may potentially contribute to staff shortages.

While there is a growing consensus regarding the need for intervention to support the redeployed and areas for attention, a major challenge is that the science of ‘what works’ regarding good-practice solutions remains under-researched, leaving employers to find their own solutions. The need for this presents as particularly acute in the context of growing policy interest in technological solutions designed to produce more agile care delivery, where greater flexibility over staff deployment is viewed as a key feature.^9 32 33^ In extremis, this could herald a cultural shift to an algorithm-determined internal labour market, in which staff have little control over when or where they work, with data-driven, ad hoc, fluid, just-in-time deployment.^34 35^

The amassed evidence pointing to the amplified vulnerability of redeployed staff suggests that policymakers should explore this issue more deeply, specifically to determine definitively whether the feature of redeployment is causal, plausibly as a component of the annual NHS staff survey.^36^ Subsequent steps could include identifying and developing suitable risk mitigation measures (eg, enhanced support for redeployed personnel), with a view to defining employer good practice.

## Limitations

Our findings show strong alignment with published evidence on the impacts associated with redeployment but are possibly unique in being based on a sample sufficient to support statistical comparison with non-redeployed staff.

Our finding of contrasts in staff-reported impacts on their health and well-being emerged as a feature of our wider research on staff retention. It suggests a need for further dedicated research to explore the implications for staff and service delivery, to answer questions that our data cannot. Specifically, our sample could not support the exploration of the implications of the frequency of redeployment, structural and demographic contrasts (eg, care-service type, occupational/profession, grade, tenure, age, ethnicity or the presence of potential confounding or intervening variables). For example, if junior early-career staff are more likely to be redeployed, this may be a contributory factor in the higher background exit rates of early-career health professionals compared with established staff. Further research is needed to explore these and other relevant variables with a sample of sufficient magnitude to support multivariate analysis to test for the independent effect of redeployment and to determine the relative strength of variables that impact staff health, well-being and stay-leave behaviour.

In common with other researchers, the absence of any published preCOVID-19 redeployment rates meant that we were unable to formally test the issue of whether postpandemic rates are above historical norms. Notwithstanding this, the finding of amplified vulnerability of redeployed staff with respect to their health, well-being and disposition to remain in NHS employment points to the need for employer action to mitigate negative impacts on staff, service providers and service users.

Our findings contribute to the growing evidence of negative impacts associated with the experience of redeployment and indicate variables that present as causal, with implications for ameliorative interventions. However, they do not extend to the identification of effective intervention measures; this logically constitutes a topic for further examination.

### Recommendations

- Policymakers and employers need to consider and further explore the indication of detrimental impacts on the health, well-being and retention of redeployed staff.
- Further research is needed to explore more deeply the health, well-being and staff retention implications of redeployment, to produce a more nuanced understanding of relevant variables, their relative impacts and how these might vary across different segments of the NHS workforce.
- Further research is needed to map the scope for intervention to effectively mitigate negative impacts associated with the redeployment on staff identified within the growing body of evidence on this issue.
- Employers should consider the scope for additional support for redeployed staff to mitigate risk factors, notably with respect to social isolation and line manager relationship.
- Institutional estimates of productivity gains from increased flexibility in the deployment of staff need to take account of the potential for higher sickness absence and leaver rates.

## Conclusions

The deployment of staff to configurations aligned with the profile of need and demand for patient care benefits from a strong intuitive appeal from the perspectives of efficiency and flexibility in dealing with change and uncertainty, such as seasonal fluctuations in demand for care and outbreaks of disease.^35^ Our findings contribute to the growing evidence of negative impacts associated with the experience of redeployment and implications for staff health, well-being and disposition to remain employed in healthcare. While the promise of greater flexibility in staff deployment affords the promise of efficiency gains, this does not present as a zero-cost option. While there is a growing consensus regarding the need for intervention to support the redeployed, a major challenge for employers is that evidence regarding ‘what works’ remains under-researched.

## Supplementary material

10.1136/bmjopen-2025-107785 online supplemental file 1

10.1136/bmjopen-2025-107785 online supplemental file 2

10.1136/bmjopen-2025-107785 online supplemental file 3

10.1136/bmjopen-2025-107785 online supplemental file 4

## Data Availability

Data are available upon reasonable request.
