# Supplementary material for: Impact of redeployment on healthcare staff well-being and retention: a survey of staff in the UK National Health Service
Source: BMJ Open. 2026 Feb 2;16(2):e107785. doi: 10.1136/bmjopen-2025-107785 (PMC12878377; doi:10.1136/bmjopen-2025-107785)
Supplement: online supplemental file 1 [file bmjopen-16-2-s001.docx]

**Appendix 1** Redeployed and Non-redeployed ratings of stress, morale, ability to switch off at home, and extent to which I enjoy my job - Wave 4

| Wave 4 (Scale 1- 5; low – high) | Redeployed (N 389) | | Non-redeployed (N1223) | | df 1590 |  |  |
| --- | --- | --- | --- | --- | --- | --- | --- |
|  | Mean | SD | Mean | SD | t | *p* | Cohen  d |
| *Morale* | 3.80 | 0.97 | 3.55 | 0.88 | 4.67 | < 0.0001 | 0.27 |
| Stress | 3.77 | 0.94 | 3.61 | 0.83 | 3.14 | 0.0017 | 0.18 |
| *Ability to switch off at home*, | 3.59 | 0.92 | 3.41 | 0.84 | 3.53 | 0.0004 | 0.32 |
| *Extent to which I enjoy my job* | 3.70 | 0.91 | 3.42 | 0.84 | 5.50 | < 0.0001 | 0.32 |
