# Supplementary material for: Impact of redeployment on healthcare staff well-being and retention: a survey of staff in the UK National Health Service
Source: BMJ Open. 2026 Feb 2;16(2):e107785. doi: 10.1136/bmjopen-2025-107785 (PMC12878377; doi:10.1136/bmjopen-2025-107785)
Supplement: online supplemental file 2 [file bmjopen-16-2-s002.docx]

**Appendix 2** Redeployed and Non-redeployed ratings of burnout - Wave 3 and Wave 4.

| Burnout Symptom  Wave 4 (Scale 1 -5; low-high) | Redeployed (N 389) | | Non-redeployed (N1265) | | df 1652 | | |
| --- | --- | --- | --- | --- | --- | --- | --- |
|  | Mean | SD | Mean | SD | t | *p* | Cohen  d |
| Overwhelmed | 3.05 | 1.09 | 2.69 | 1.10 | 4.65 | < 0.0001 | 0.32 |
| Low energy | 3.24 | 1.10 | 3.04 | 1.12 | 2.97 | <0.0001 | 0.18 |
| Negative feelings | 3.03 | 1.17 | 2.74 | 1.15 | 4.39 | < 0.0030 | 0.25 |
| Disconnected | 2.68 | 1.09 | 2.37 | 1.13 | 4.70 | < 0.0001 | 0.28 |
| Ineffective | 2.78 | 1.07 | 2.44 | 1.08 | 5.60 | < 0.0001 | 0.33 |
| Physical exhaustion | 3.13 | 1.12 | 2.70 | 1.15 | 6.50 | < 0.0001 | 0.36 |
| Mental Exhaustion | 3.20 | 1.18 | 2.82 | 1.15 | 5.71 | < 0.0001 | 0.33 |
| Dread going to work | 2.80 | 1.181 | 2.34 | 1.17 | 6.75 | < 0.0001 | 0.39 |
| Feel helpless | 3.49 | 1.12 | 3.20 | 1.13 | 3.53 | < 0.0001 | 0.26 |
| Loss of empathy with patients | 2.75 | 1.17 | 2.27 | 1.18 | 6.93 | < 0.0001 | 0.40 |

| Burnout Symptom  Wave 3 (Scale 1 -5; low – high) | Redeployed (N 354) | | Non-redeployed (N1209) | | df 1561 | | |
| --- | --- | --- | --- | --- | --- | --- | --- |
|  | Mean | SD | Mean | SD | t | *p* | Cohen  d |
| Overwhelmed | 2.77 | 1.13 | 2.63 | 1.08 | 2.08 | 0.0378 | 0.13 |
| Low energy | 3.11 | 1.05 | 2.96 | 1.06 | 2.30 | 0.0218 | 0.14 |
| Negative feelings | 2.86 | 1.11 | 2.72 | 1.12 | 2.03 | 0.0427 | 0.13 |
| Disconnected | 2.51 | 1.08 | 2.28 | 1.09 | 3.42 | 0.0006 | 0.21 |
| Ineffective | 2.51 | 1.11 | 2.36 | 1.07 | 2.25 | 0.0245 | 0.14 |
| Physical exhaustion | 2.87 | 1.08 | 2.68 | 1.11 | 2.78 | 0.0054 | 0.17 |
| Mental Exhaustion | 2.92 | 1.11 | 2.74 | 1.14 | 1.49 | 0.1378 | - |
| Dread going to work | 2.57 | 1.26 | 2.36 | 1.17 | 2.86 | 0.0043 | 0.17 |
| Feel helpless | 3.21 | 1.07 | 3.12 | 1.07 | 0.77 | 0.4428 | - |
| Loss of empathy with patients | 2.38 | 1.11 | 2.22 | 1.11 | 2.34 | 0.0197 | 0.14 |
