# Supplementary material for: Impact of redeployment on healthcare staff well-being and retention: a survey of staff in the UK National Health Service
Source: BMJ Open. 2026 Feb 2;16(2):e107785. doi: 10.1136/bmjopen-2025-107785 (PMC12878377; doi:10.1136/bmjopen-2025-107785)
Supplement: online supplemental file 3 [file bmjopen-16-2-s003.docx]

**Appendix 3** Redeployed and Non-redeployed ratings of worry - Wave 4

| Ratings of Worry  Wave 4 (Scale 1-10; low-high) | Redeployed (N 389) | | Non-redeployed (N1265) | | df 1854 | | |
| --- | --- | --- | --- | --- | --- | --- | --- |
|  | Mean | SD | Mean | SD | t | *p* | Cohen  d |
| Impact on my mental health | 7.11 | 2.51 | 6.30 | 2.74 | 4.32 | < 0.0001 | 0.30 |
| Impact on my physical health | 6.70 | 2.59 | 5.78 | 2.80 | 4.88 | *< 0.0001* | 0.31 |
| Being given too much responsibility | 4.39 | 2.72 | 3.98 | 2.62 | 2.22 | 0.0265 | 0.15 |
| Being asked to do work I have not been trained for | 5.14 | 2.91 | 4.34 | 2.90 | 4.01 | *< 0.0001* | 0.28 |
| Not having enough time to do my job properly | 6.22 | 2.89 | 5.44 | 2.92 | 3.87 | < 0.0001 | 0.27 |
| Making mistakes because of my workload | 5.71 | 2.81 | 5.29 | 2.83 | 2.15 | 0.0317 | 0.15 |
| Abnormally high staff shortages | 7.41 | 2.41 | 6.52 | 2.72 | 4.99 | < 0.0001 | 0.35 |
| Colleagues lacking necessary skills and competencies | 6.59 | 2.79 | 5.91 | 2.78 | 3.54 | 0.0004 | 0.25 |
| Lack of support from my line manager | 5.01 | 3.03 | 4.27 | 2.92 | 3.64 | *0.0003* | 0.25 |
| The impact on the NHS of removing all COVID restrictions | 4.31 | 2.95 | 3.88 | 2.80 | 2.20 | *0.0284* | 0.15 |
| Dealing with waiting lists for non-CCOVID treatment | 5.33 | 3.27 | 4.23 | 3.06 | 5.16 | *< 0.0001* | 0.35 |
| No having any say about being redeployed to a different role or team | 5.59 | 3.29 | 4.05 | 3.15 | 7.03 | *< 0.0001* | 4.78 |
| The NHS will be able to handle future pandemics effectively | 7.50 | 2.54 | 7.01 | 2.85 | 2.58 | *0.0100* | 0.18 |
| My financial wellbeing | 7.45 | 2.70 | 6.78 | 2.80 | 3.52 | *0.0004* | 0.24 |
| The risk of being blamed personally for poor care | 6.04 | 3.02 | 5.52 | 3.10 | 2.47 | *0.0136* | 0.17 |
| Aggression from patients or the public | 5.21 | 2.88 | 4.84 | 2.91 | 1.87 | *0.0596* | - |
