# Supplementary material for: Impact of redeployment on healthcare staff well-being and retention: a survey of staff in the UK National Health Service
Source: BMJ Open. 2026 Feb 2;16(2):e107785. doi: 10.1136/bmjopen-2025-107785 (PMC12878377; doi:10.1136/bmjopen-2025-107785)
Supplement: online supplemental file 4 [file bmjopen-16-2-s004.docx]

**Appendix 4** Wave 4 Survey

**Your Future in the NHS**

**Wave 4 questionnaire v8 – 08-03-23 (for main launch)**

QS. Are you… (CODE ALL THAT APPLY)

A direct employee of the NHS in England (i.e. working for a Trust or another NHS body) – CONTINUE

An employee of an organisation that supplies patient care services to the NHS – CLOSE

An employee of an organisation that provides non–patient care services to the NHS e.g. estates; domestic; technical services or IT – CLOSE

An employee in public health or OHID in England – CLOSE

Working for a GP surgery – CLOSE

Employed by a nursing agency or nursing bank – CLOSE

Working in a non-NHS care or nursing home – CLOSE

Working in some other setting – CLOSE

Q1. What is your occupational group? If your group isn’t shown below, please select ‘Other’ and enter the details there.

**Administrative Management**

**Admin & Clerical** (including Medical Secretary)

**Allied Health**

- Professionals (e.g. physiotherapy, psychotherapy; occupational therapy, dietetics, speech and language therapy, complementary therapy; art therapy)
- Allied Health non-registered or other (e.g. support worker, therapy helper, therapy assistant or student)

**Ambulance**

- Students and newly qualified paramedics
- Paramedic
- Emergency Medical Technician/Emergency Care Assistant
- Call centre staff 999
- Call centre staff 111
- Patient Transport Service
- Ambulance service registered clinicians (non- Paramedic)
- Manager or office-based roles

**Dental**

**Estates, ancillary and support staff (e.g. porter; catering; maintenance cleaning)**

**Central technical and administrative /Corporate services (e.g. Finance and IT)**

**Clinical Management**

**Clinical Psychology**

**Commissioning Managers**

**Medical**

- Consultant
- In training (e.g. Foundation year 1 & Y2 including FTSTAs & LATs, SHOs, SpRs, SpTs, GPRs)
- Medical/dental – other (e.g. staff and associate specialists or non-consultant career grade)

**Midwifery**

- Midwife
- Maternity Support Worker

**Nursing (registered)**

- Hospital – emergency, critical care, theatre
- Hospital – adults or children
- district/community including learning disability and mental health
- mental health and learning disability (non-community)
- Other Registered Nurses (e.g. 999, 111)

**Nursing support** (including Auxiliary/Nursing Assistant/Nursing Associate/Healthcare Assistants/health, clinical and nursing support workers)

**Pharmacy**

**Research**

**Scientific and technical**

- professional (e.g. haematology, clinical, biochemistry, microbiology)
- support.

**Social Care**

- Manager
- Social worker
- Social Care Support Staff

Other (PLEASE SPECIFY)

Q2. Do you have formal responsibility for supervising or line managing other NHS staff? MULTICODE OKAY FOR THE YES CODES

Yes, supervising

Yes, line managing

No

Q3. In total, how many years (to the nearest full year) have you worked in the NHS? Please include any parental leave but exclude career breaks. If you have worked for less than a year, please type in 0.

ENTER NUMBER

Q4a. What type of NHS service do you work in?

Mental Health

Acute Hospital including specialist Trusts (e.g. for children, cancer, neuro)

Community Health

Ambulance

Other type of service, e.g. NHS England, ICS, ICP (PLEASE SPECIFY)

ASK ALL BAR NHS TRUST SAMPLES

Q5. What is the name of the NHS Trust or other NHS organisation that you work for?

VERBATIM with Prefer not to say option

ASK ALL

Q6. How long have you been working for your current NHS Trust/organisation?

Under 6 months - SKIPS Q12

At least 6, up to 12 months

At least 1, up to 2 years

At least 2, up to 5 years

At least 5 years

Q7. SKIPPED

Q8a. What was your age last birthday?

ENTER NUMBER (18+) or if PNTS, offer these range options.

18-24

25-29

30-34

35-39

40-44

45-49

50-54

55-59

60-64

65+

Q8b. Do you identify as…

Male

Female

In another way (PLEASE SPECIFY)

Prefer not to say

Q9. What ethnic group best describes you? Please select one option only.

**White**

1. English/Welsh/Scottish/Northern Irish/British

2. Irish

3. Gypsy or Irish Traveler

4. Any other White background, please describe

**Mixed/Multiple ethnic groups**

5. White and Black Caribbean

6. White and Black African

7. White and Asian

8. Any other Mixed/Multiple ethnic background, please describe

**Asian/Asian British**

9. Indian

10. Pakistani

11. Bangladeshi

12. Chinese

13. Any other Asian background, please describe

**Black/ African/Caribbean/Black British**

14. African

15. Caribbean

16. Any other Black/African/Caribbean background, please describe

**Other ethnic group**

17. Arab

18. Any other ethnic group, please describe

Prefer not to say

Q10. Are your day-to-day activities limited because of a health problem or disability which has lasted, or is expected to last, at least 12 months?

- Yes, limited a lot
- Yes, limited a little
- No
- Prefer not to say

ASK ALL BAR MEDICS AT Q2

Q11. What is your job band/grade?

Band 1

Band 2

Band 3

Band 4

Band 5

Band 6

Band 7

Band 8a

Band 8b

Band 8c

Band 8d

Band 9

Senior management

Other (PLEASE SPECIFY)

ASK FOR MEDICAL ONLY (Q1)

Q11b. What is your job grade?

FY1/HO1

FY2/HO2

SHO

StR CT1-2

StR CT3-5

ST1-2 / SpR1-2

ST3-5 / SpR3-5

ST8 / SpR6-10

Junior clinical fellow

Senior clinical fellow

Senior clinician/consultant

Other (PLEASE SPECIFY)

**Your experiences at work**

ASK ALL WHO HAVE BEEN WORKING FOR THEIR ORGANISATION FOR AT LEAST 6 MONTHS at Q6

Q12. Please now think about the 6-month period since September 2022. In your organisation, for each of the following, has the situation got better, got worse or is it unchanged?

A lot better, better, unchanged, worse, a lot worse

**Recognition of my contribution by…**

- The public
- The government
- My employer
- My line manager

**Support for me from…**

- My employer
- My line manager
- My colleagues

**My well-being:**

- My level of morale
- My mental health
- My physical health
- My stress levels
- My ability to switch off from work when home.

**My job**

- My workload
- The amount of paid overtime I work N/A CODE
- The amount of unpaid overtime I work
- The availability of equipment and resources needed to do my job
- Staffing levels
- The extent to which I enjoy my job
- My satisfaction with the standard of care I am able to give to patients or service users (with N/A option)

ASK ALL

Q13 Thinking about the current situation, to what extent are the following a source of worry for you? [1-10 scale – anchored with ‘not at all worried’ through to ‘extremely worried’] STATEMENTS ROTATED

Not having any say about being redeployed to a different role or team

The NHS being able to handle future pandemics

The impact of my work on my mental health

The impact of my work on my physical health

Being given too much responsibility

My financial well-being

Being asked to do work I have not been trained for

Not having enough time to do my job properly

Making mistakes because of my workload

Abnormally high staff shortages

Colleagues lacking necessary skills and competencies

Lack of support from my line manager

The impact on the NHS of removing all COVID restrictions

Dealing with the waiting lists for non-COVID treatment

Risk of being blamed personally for poor care (professionals only)

Aggression from patients or the public

ASK FOR EACH SCORED AT 6+ AT Q13

Q14 Have you raised your worries about (INSERT ISSUE) with your line manager

1. Yes, I raised my worries
2. No – didn’t raise my worries

Q14b ASK THOSE WHO HAD HIGH LEVEL OF WORRY (6+) BUT DID NOT RAISE IT WITH LINE MANAGER (CAPPED AT ONE PER INTERVIEW, SELECTED RANDOMLY IF 2+ ELIGIBLE)

You rated X as a major worry with a score of Y but haven’t raised it with your line manager. Please tell us why you have not discussed this with your line manager. PLEASE CODE ALL THAT APPLY. LIST RANDOMISED

No point – nothing happens

External issue that my manager can’t address

Manager is already aware

Don’t believe line manager would be supportive

Lack of contact with line manager

Lack of rapport/poor relationship with line manager

We are all in the same boat

Would make it look like I’m not capable of doing my job

Don’t want to put more pressure on line manager

Would be labelled as a trouble-maker

Haven’t got round to raising the issue yet

Prejudice/discrimination

Other (PLEASE SPECIFY)

ASK ALL

Q15 We are interested in your motivation to work in the NHS. Please select up to **three** reasons that keep you working for the NHS. If some of your views are not shown in the list here, please select ‘Other’ and enter the details there. RESPONSES ROTATED

Being appreciated by patients or service users

Being appreciated by the public

Being appreciated by my manager

Being appreciated by my employer

Personal commitment to working for the NHS

To make a difference

Job satisfaction from caring for patients or service users

The people I work with

Pay - relative to alternative employment

Career or promotion opportunities

Opportunities for flexible or part-time working

Lack of other (non-NHS) employment options.

Job security

NHS pension

Other (PLEASE SPECIFY)

None of the above – I would like to leave the NHS

Q16 How important are each of these reasons to explain why (INSERT HIGH LEVEL OCCUPATIONAL GROUP FROM Q1 UNLESS ‘OTHER’) staff leave the NHS? RESPONSES ROTATED

Very, fairly, not very, not at all important

Workload

Shortages of staff/resources

Working hours

Stress

Impact on mental health

Contribution not being recognised by employers

Being undervalued by government

Being undervalued by line managers

Being undervalued by senior managers

Lack of opportunity for flexible or part-time working

Unsupportive managers

Bullying

Pay

Red tape and bureaucracy

Impact on physical health

Ability to provide good patient/service user care

Career or promotion opportunities

Discrimination or prejudice

Aggression from patients or public

YOUGOV LONGITUDINAL SAMPLE ONLY

Q16b. IDENTIFY THOSE WHOSE ANSWER SHIFTED BY 2+ POINTS IN EITHER DIRECTION SINCE LAST INTERVIEW (WHETHER WAVE 1 OR WAVE 2) AND SELECT ONE AT RANDOM IF MORE THAN ONE HAS MOVED. ONLY APPLIES TO VARIABLES USED AT EACH WAVE

When you answered the question about A some months ago, you gave an answer of X while now you said Y. Is there any particular reason why you have changed your views? VERBATIM

Q17. To what extent do you agree/disagree with the following statements?

Strongly agree

Agree

Neither agree nor disagree

Disagree

Strongly disagree

I would never want to work outside the NHS

I would recommend working for the NHS to others

ASK ALL

Q18a. During the last 6 months, have you: (NO ROTATION)

Yes

No

- Talked to colleagues and/or former colleagues about job opportunities outside the NHS
- Actively looked at vacancy lists for jobs outside the NHS
- Requested details of other job(s) outside the NHS but decided against applying
- Submitted job applications for jobs outside the NHS
- Been interviewed for jobs outside the NHS
- Been offered a job outside the NHS

IF HAD DONE SOMETHING BEYOND TALKING TO COLLEAGUES, UNLESS OFFERED A JOB IS THE ONLY CODE IN WHICH CASE GO TO Q19

Q18b Which of these have influenced you to look for work outside of the NHS in the last 6 months? Please select up to three reasons. If some of your views are not shown in the list here, please select ‘Other’ and enter the details there. RESPONSES ROTATED

Worry over the risk to my health of catching COVID 19

Worry over the risk of my infecting others with COVID 19

Treatment by Government

Treatment by employers

Treatment by line managers

Lack of career or promotion opportunities

Wanting higher pay

Excessive workload

Dissatisfaction with the standard of care I am able to give to patients/service users

Poor work-life balance

Lack of opportunity for flexible or part-time working

Patterns of working hours (shift pattern or length)

Amount of unpaid overtime

The impact of my work on my mental health

The impact of my work on my physical health

Lack of resources (staff/equipment)

Bullying

Discrimination or prejudice

Need additional income from second job, on top of my NHS role

Other [PLEASE SPECIFY)

IF APPLIED FOR A NON-NHS JOB IN LAST 6 MONTHS AT Q18

Q18c How many non-NHS job applications have you made in the last 6 months?

1

2

3

4

5+

Q18d What was the last job (outside the NHS) that you applied for?

VERBATIM

Q18e Would this job have been in addition to your NHS role or to replace it?

In addition

To replace it

IF HAVE BEEN OFFERED A NON-NHS JOB IN THE LAST 6 MONTHS AT Q18

Q18f Have you accepted the non-NHS job you were offered most recently?

Yes

No

Not decided yet

IF HAVE NOT ACCEPTED THE JOB

Q18g Why did you decide not to accept this job offer?

VERBATIM

ASK ALL

Q19 Which of the following best describes what you would like to be doing in five years from now? If some of your views are not shown in the list here, please select ‘Other’ and enter the details there.

Continue working for the NHS

Move to bank/agency work

Get a job in private sector health care

Get a job outside the health care sector/become self-employed

Retirement – no longer in paid work

Career break

Other (PLEASE SPECIFY)

No plans – I have not thought about this

Working Patterns

ASK ALL

Q20a. In the last 6 months, have you taken on extra hours of paid work because of the rising cost of living?

Yes – with my NHS employer

Yes – health sector bank or agency work

Yes – other non-NHS employment

No

ASK ALL

Q21. In the last six months, have you experienced abnormal pressure from your NHS employer to work more hours? ROTATE ORDER OF CODES

A lot

A fair amount

A little

None at all

Qs 22-23 omitted.

ASK ALL

Q24. Since March 2020, have you worked in areas where there were patients diagnosed with COVID-19?

Yes, frequently

Yes, occasionally

No, never

Don’t know

Q25a. In the last 6 months, how often if at all have you been redeployed to a different location, department or team?

Never

Occasionally

Often

ASK FOR OFTEN/OCCASIONALLY

Q25c. Was your most recent redeployment voluntary or involuntary?

Voluntary

Involuntary

Q 26. Thinking about the next 12 months, how confident are you about the following issues? ROTATE ORDER OF STATEMENTS

Very, fairly, not very, not at all

- The NHS will get the funding resource it needs
- Your organisation will proactively support your health and wellbeing
- The vaccine programme will be effective in controlling COVID 19.
- My daily workload will go down
- My stress levels will go down
- We have seen the worst of the COVID-19 pandemic
- Staffing levels in my Trust or NHS organisation will improve
- My own future working in the NHS
- The NHS will be able to cope with the demand for non-COVID-19 healthcare
- NHS resources will be prepared for a further wave of the COVID-19 pandemic
- I will be satisfied with the standard of care I am able to deliver (include a n/a option)
- Staff in your team will stay working in the NHS
- The NHS organisation I work for will be able to deliver an acceptable standard patient care (include a n/a option)
- I will be working with people that I know on my next shift.

Q27. Over the last six months, to what extent have you experienced the following? RANDOMISE ORDER OF ISSUES.

Never/occasional days/some days/most days/every day

- Feeling overwhelmed
- Low energy
- Negative feelings
- Feeling disconnected from my work
- Feeling professionally ineffective
- Physical exhaustion
- Mental exhaustion
- Dreading going to work
- Feeling very tired or drained
- Feeling helpless
- Losing empathy with patients or service users

IF EVERY DAY/MOST DAYS – PICK ONE AT RANDOM FOR Q7

Q28. Thinking about … to what extent do you attribute this to your job in the NHS?

Completely/partly/not at all

Q29. Thinking about the last 6 months, were there any occasions when you worked when you were really too ill and should have taken sick leave?

Yes

No

IF YES

Q30. Were you suffering from… (CODE ALL THAT APPLY)

- An injury or other physiological health issue e.g. bad back, or long term condition.
- COVID
- A (non-COVID-19) viral infection such as flu
- A mental health issue
- Another health issue (PLEASE SPECIFY)

Q31. Thinking about the most recent occasion you worked while ill during the last 6 months. To what extent did the following influence your decision to work when you were ill and should have taken sick leave? ROTATE ORDER OF ITEMS

Strongly influenced/had some influence/had no influence

- Losing pay
- Your manager’s reaction
- Falling behind with your work
- Extra burden placed on colleagues
- Letting patients/service users down
- No-one else could cover your role
- Exceeding your sickness-absence days allowance
- Getting a bad reputation
- Impact on your attendance record

ASK ALL

Q32. We are interested in your views on priorities for change that would improve retention rates for (INSERT HIGH LEVEL OCCUPATIONAL GROUP) staff in the NHS. Please select up to three priorities for change/improvement from the list below **(if you select** ‘Other’, please give details). RESPONSES ROTATED

Greater recognition by my line manager

Greater recognition by senior managers

Greater recognition by the government

Greater support from line managers

Greater support from senior managers

Increase in (non-staff) resource levels

Increase in staffing levels

Pay – rate relative to alternative non-NHS employment

More opportunity for flexible or part-time working

Reduced workload

Improved career/promotion opportunities

Changes to working hours/shift patterns

Reduced patient/service user waiting times

Opportunities to take breaks/eat/drink

Other (PLEASE SPECIFY)

Q33. Is there anything you would like to add in relation to any of the issues addressed in this questionnaire? VERBATIM

List of alterations to Survey Question by Wave.

| **Changes Wave1-2:** | |
| --- | --- |
| **Added – Q16**_16 Discrimination or prejudice | Reason to leave [scale 1-10] |
| **Added – Q16**_17 Discrimination/ prejudice |  |
| **Added Q19c_7** Working directly for the NHS | Rating scale |
| **Omitted – [Q20a]** How many hours a week are you contracted to work excluding overtime? | [1] At least 37.5 hours a week [2] 30-37 hours a week[3] Under 30 hours a week |
| **Added [Q20]** Are you currently working more hours each week (including paid or unpaid overtime), compared with the hours you worked _before March 2020? | \| Yes – now work more hours \| \| --- \| \| No – not working more hours \| \| Don’t know \| |
| **Omitted [Q20b] {single}** How many hours did you work in your last shift/working day? | [1] Less than 8 hours [2] 8, up to 10 hours  [3] Over 10 up to 12 hours [4] Over 12 hours [5] Not applicable |
| **Added [Q21]** Since March 2020 have you experienced any pressure from your employer to work more hours? | \| A lot \| \| --- \| \| A fair amount \| \| A little \| |
| **Omitted [Q22]** How many hours unpaid overtime did you work in your last shift/working day? | 1] 0 hours [2] 1-2 hours [3] 3-5 hours [4] Over 6 hours [5] |
| **Omitted at Wave 2 [Q23]** Do you regularly work (night shifts) between the hours of 7 pm and 7 am? | [1] Yes [2] No |
| **Added** [Q25c] And was your redeployment to work with COVID-19 patients voluntary or involuntary? | \| <1> \| Voluntary \| \| --- \| --- \| \| <2> \| Involuntary \| |
| **Added** [Q28] Are you a member of a trade union or a member of a professional body | [1] Trade union; [2] Professional body, [3] No |
| **Added** [Q29a] Thinking about the period since the mass outbreak of COVID-19 in March 2020 – using the 7-point scale provided, how would you **rate your line manager** on each of the following  [Q29b} …. rate the NHS organisation you work for** on each of the following?  [Q29c] **….** rate the Government** on each of the following? | Ignoring \| Caring; Insensitive \| Sensitive; Inconsiderate \| Considerate; Dishonest \| Honest; Unbelievable \| Believable; Untruthful \| Truthful; Incompetent \| Competent; Unqualified \| Proficient; Incapable \| Capable; Clueless \| Knowledgeable; Irresponsible \| Responsible; Unaccountable \| Accountable |
|  | |
| **Changes at Wave 3** |  |
| **Added** [Q13_16] The impact on the NHS of removing all COVID restrictions | [Worry scale 1- 10] |
| **Added** [Q13_17] Dealing with the waiting lists for non-COVID treatment | [Worry scale 1- 10] |
| **Omitted** Q28 Are you a member of a trade union or a member of a professional body | (see Table 1 |
| **Omitted** [Q29a;b & c**]** Thinking about the period since the mass outbreak of COVID-19 in March 2020 – using the 7-point scale provided, how would you **rate your line manager** on each of the following? | (see Table 1) |
| **Added** [Q34] How many COVID-19 vaccinations have you had | 0 – none, 1, 2, 3, prefer not to say |
| **Added** [Q34b] Why have you chosen not to be vaccinated? | Open end |
| **Added [Q24c]** Were you happy or reluctant to be vaccinated | Very happy; Fairly happy; Neither; Fairly reluctant; Very reluctant |
| **Added [Q34d]** You said earlier that you have applied for a job outside the NHS in the last 6 months. Did you do this because you might have been faced with losing your NHS job because of the vaccination issue | [1] Yes‘ [2] No |
| **Added [Q33]** Thinking more generally about the next 12 months, how confident are you about the following issues  The NHS will get the funding resource it needs  Your organisation will proactively support your health and wellbeing  The vaccine programme will be effective in controlling COVID 19  My daily workload will go down  My stress levels will go down  We have seen the worst of the COVID-19 pandemic  Staffing levels in my Trust or NHS organisation will improve  My own future working in the NHS  The NHS will be able to cope with the demand for non-COVID healthcare  NHS resources will be prepared for a further wave of the COVID-19 pandemic  I will be satisfied with the standard of care I am able to deliver  Staff in your team will stay working in the NHS | <1> Very confident  <2> Fairly confident  <3> Not very confident  <4> Not at all confident |
| **Added [**Q35] Over the last six months, since October 2021, to what extent have you experienced the following?  Feeling overwhelmed  Low energy  Negative feelings  Feeling disconnected from my work  Feeling professionally ineffective  Physical exhaustion  Mental exhaustion  Dreading going to work  Feeling very tired or drained  Feeling helpless | Never  Occasional days  Some days  Most days  Every day |
| **Added** Q[36] Thinking aboutQ35 to what extent do you attribute this to your job in the NHS? | Completely; Partly; Not at all |

**[**

| **Changes Wave 4** |  |
| --- | --- |
| **Omitted-[**Q16_1] Catching/ spreading COVID-19 | Scale  Very important  Fairly important  Not very important  Not at all important |
| **Omitted** [Q16_2] Time pressure |  |
| **Added** [Q16_17] Workload |  |
| **Added** [Q16_18] Being undervalued by government |  |
| **Added** [Q16_19] Being undervalued by line managers |  |
| **Added** [Q16_20] Being undervalued by senior managers |  |
| **Added** [Q16_21] Aggression from patients or public |  |
| **Omitted** **[**Q17] I would never want to work outside the NHS | \| Strongly agree; \| \| --- \| \| Agree; \| \| Neither agree nor disagree \| \| Disagree \| \| Strongly disagree \| |
| **Added** [Q18c] How many non-NHS job applications have you made in the last 6 months? | 1;2;3;4;5+ |
| **Added** [Q18d] What was the last job (outside the NHS) that you applied for? | Open |
| **Added** [Q18e] Would this have been in addition to your NHS role or to replace it | <1> In addition; <2>To replace it |
| **Added** [Q18f] Have you accepted the non-NHS job you were offered most recently | <1> Yes; <2> No‘ <3> Haven’t decided yet |
| **Added** [Q20a] In the last 6 months have you taken on extra hours of paid work because of the rising cost of living | Yes, with my NHS employer; Yes, health sector bank/ agency work; Yes, other non-NHS employment; No |
| **Omitted** [Q20b] Are you now working more or fewer hours than you were 12 months ago, or is it about the same? | More; About the same; Fewer |
