# Supplementary material for: Impact of redeployment on healthcare staff well-being and retention: a survey of staff in the UK National Health Service
Source: BMJ Open. 2026 Feb 2;16(2):e107785. doi: 10.1136/bmjopen-2025-107785 (PMC12878377; doi:10.1136/bmjopen-2025-107785)
Supplement: Supplementary file 5 [file bmjopen-2025-107785.reviewer_comments.pdf]

PEER REVIEW HISTORY

BMJ Open publishes all reviews undertaken for accepted manuscripts. Reviewers are asked to complete a checklist review form and are provided with free text boxes to elaborate on their assessment. These free text comments are reproduced below.

ARTICLE DETAILS

Title (Provisional)

The impact of redeployment on healthcare staff wellbeing and retention: A survey of staff in the UK National Health Service.

Authors

Weyman, Andrew; Glendinning, Richard; O'Hara, Rachel

VERSION 1 - REVIEW

**Reviewer**	**1**
**Name**	**Baek, Eun-Mi**
**Affiliation**	**The Catholic University of Korea, Department of Preventive Medicine**
**Date**	**14-Sep-2025**
**COI**	**None**

I appreciate the opportunity to review good manuscripts prior to publication. I hope you find my review helpful.

The primary strength of this study lies in its utilisation of large-scale panel data. With over 1,500 participants in each survey wave, the sample size was sufficiently large and possessed characteristics representative of NHS staff. Furthermore, data collection across four time points—from the early pandemic to the post-pandemic period—enabled observation of temporal changes, facilitating trend-based analysis rather than merely cross-sectional findings.

The study significantly enhanced generalisability by encompassing diverse groups, including all major NHS job categories: nursing, medical, emergency, and administrative. Crucially, unlike many previous studies confined to a single job category, securing this occupational diversity represents a major advantage.

This study primarily relied on simple comparative statistics such as t-tests and Z-tests, which presents a clear limitation in that it did not adequately control for key confounding variables such as age, gender, tenure, job grade, and occupational group. As a result, the relationships

between redeployment, burnout, and turnover intention are demonstrated only at the level of simple associations, without fully disentangling their independent effects.

Future research would benefit from the following methodological enhancements:

Strengthening control for confounding variables

Rather than relying solely on simple group comparisons, multivariate regression analyses should be employed to simultaneously adjust for age, gender, tenure, job grade, and occupational group. This would allow for a clearer identification of the independent effect of redeployment.

Utilizing longitudinal data

Although the study collected data across four waves (2020–2023), the analysis was largely limited to cross-sectional comparisons. Leveraging the longitudinal design by applying repeated-measures ANOVA or mixed-effects models would allow researchers to explore temporal trends and interaction effects of redeployment over time, thereby strengthening both the explanatory power and persuasiveness of the findings.

Deepening causal exploration

While definitive causal inference is not possible, structural equation modeling (SEM) or path analysis could be applied to examine indirect and mediating effects, such as "redeployment → increased stress → burnout → turnover intention." Such an approach would provide richer interpretation beyond mean differences and help to clarify the pathways through which redeployment influences workforce outcomes, offering more practical insights for workforce management and policy interventions.

In sum, authors should be cautious not to overgeneralize the implications of the current analytical approach. Future studies should prioritize methodological advancements that strengthen confounder control, capitalize on the longitudinal nature of the data, and employ causal modeling strategies to deepen understanding of the mechanisms linking redeployment to staff outcomes.

**Reviewer**	**2**
**Name**	**Rippon, Daniel**
**Affiliation**	**Northumbria University**
**Date**	**10-Nov-2025**
**COI**	**None**

Abstract

In the summary of the method, could you please summarise the type of statistical analyses that were conducted to analyse the quantitative data.

Please correct the spelling mistake of presentism, I assume that you mean presenteeism here.

Strengths and Limitation

Note the misspelling ‘quantitive’.

Please address the following: “This paper focuses on the most recent wave of the survey, due to is contemporary relevance and the post-pandemic return to normal care.

Please check some of the typos and errors throughout.

Introduction

Ensure to frame the introduction with a theoretical framework that provides an explanation as to why the process of being redeployed as healthcare professional may cause staff to consider leaving healthcare professions in the NHS.

The term ‘functions’ in line 38/Introduction, should this not read as ‘services’ instead?

With regards to the following “that are experiencing shortages due to absence or non-normal”, it would be useful to precede the word absence with ‘staff’ as it currently reads as though you are referring to an absence in the demand for care.

Please support the following point with a relevant citation: “However, there are indications that the amplified rates of redeployment necessary during the COVID-19 pandemic persist and may become an increasingly encountered feature of working-life within the NHS”.

The use of the word ‘sponsored’ appears to be a little odd in the following passage “COVID-19 working conditions sponsored notable redress”. Could you please rephrase accordingly.

Please ensure to support the following point with a relevant citation: “COVID-19 working conditions sponsored notable redress in this respect, within the UK and internationally, typically as a component of broader perspectives on staff experiences, with a strong focus on the intuitively most challenging redeployments to emergency, intensive and end-of-life care.”

Could you please ensure greater clarity in the following sentence: “While findings of positive staff experiences are also reported,4; 5 a negative profile dominates author accounts by volume and emphasis”.

Ensure to clearly illustrate some of the potential benefits of being redeployed as indicated by the cited studies. The sentence above could also be reworded to ensure clarity.

Could you please write the following sentence more concisely: “The majority of studies focused on single health professions or functions and the impact of non-normal conditions during the height of the COVID-19 pandemic but share the perspective of characterising

redeployed staff as a more vulnerable "…high-risk group so should be a target for health and well-being interventions" p.37812" .

The following sentence could also be more concise or broken down into a couple of sentences: "The profile of demand for care and prevailing conditions during the pandemic is reported to have sponsored a more centralised command and control structure, particularly within secondary care, with senior managers taking a more strategic role in staff deployment decisions, supported by accelerated rates of adoption of e-rostering technology, the latter reported to have "…proven instrumental when redeploying staff,... Specific skillsets can more easily be matched to the right shifts when redeploying staff …during the COVID-19 response to redeploy staff quickly to support areas in need."16 "

The introduction as a whole is mainly focused on the redeployment of healthcare staff during the COVID-19 pandemic as a means to cover service provision. There are a couple of sentences that illustrate that the redeployment strategy employed during the COVID-19 pandemic has persisted during the post-pandemic era. However, it would be useful to review more recent studies on staff well-being in healthcare professionals beyond the COVID-19 that are relevant to the variables that were assessed in the present study. As the present study also assessed the occupational well-being of non-deployed staff, it would be useful to review literature that illustrates how stability in workplace settings could be beneficial to the welfare of healthcare professionals in secondary care services.

Method

The following sentence could be more specific in stating intentions to stay or leave a position in the NHS as a healthcare professional: "Epistemologically, the research trajectory reflected a systems perspective, focused on contextual influences on employee health, well-being and disposition to stay or leave".

The following sentence could be reworded for clarity. Terms such as 'incubating precursors' read as rather convoluted here: "Taking a risk mitigation and control perspective, the focus was on identifying associative influences that can be characterised as incubating precursors with the potential to challenge staff resilience to prevailing working conditions/arrangements and diminish their attachment to NHS employment."

What is meant by "amplified vulnerability" in the following sentence? "The core objective was to identify priority issues for intervention, nuanced by identifying the demographics and functional groups of employees that exhibited amplified vulnerability." Could a simpler term, such as 'A core objective was to assess the demographics of healthcare professionals to identify if there are particular groups in the NHS who are vulnerable to leaving their profession':

Ensure to not report results in the Method section as per the following sentence "Our initial headline analysis19; 20 revealed that redeployed staff exhibited a consistently more negative response profile on almost all issues covered by the survey".

Ensure to cite the studies/researchers that you are referring to here: "The relatively large sample allowed formal testing of associations and contrasts including those identified in (predominantly small sample; single occupation) studies by other researchers…"

The sample

Does table 1 refer to data from healthcare professionals working in England only. I.e was the data from UK services outside of England omitted from wave 1 and wave 2 for the analyses in this study?

Ensure to include a Materials subsection that details the measures that were administered to staff over the four waves and how they were used to assess the variables of interest.

Analysis

The following section is a little unclear: "The findings focus primarily on the analysis from the fourth wave of data capture as the primary focus, due to their greater contemporary relevance". Why was the focus on analysing cross sectional data at wave four. The introduction illustrates a rationale for a longitudinal assessment of the variables of interest. Yet, it is stated here that the primary focus was to assess cross-sectional data at wave four. Further clarity is required here. Could you also be more specific in this section and state the types of inferential statistical analyses that were applied in this study.

Please ensure to be specific that the following change was applied at wave 4: The reference time frame was amended to 'In the last 6 months, how often if at all have you been redeployed to a different location, department or team?' (in place of 'since March 2020', used at the three earlier waves)

Could the following sentence be stated with greater clarity: This change was considered necessary due to the increasingly distal anchor of the beginning of the pandemic, and transition to more normal working conditions and arrangements.

Results

Please ensure to state the analysis that was conducted to indicate that rates of involuntary redeployments had increased over the four waves as per the following: "At wave four, 24% of respondents said that they had been redeployed at some point in the previous six months. Of these, 25% reported this happening often and 75% occasionally. 30% of redeployments were reported to have been voluntary and 70% involuntary; with an indication of a rising rate of involuntary redeployments across the four waves" Ensure to report the inferential statistics in accordance with APA guidelines that clearly show how rates of involuntary redeployment increased over the four waves. As it is currently reported,

it is unclear if the reported increased rates of involuntary redeployment were significant or non-significant.

There does not appear to be a figure in the appendices that has been clearly labelled as Figure 1.

With regards to the section on Figure 2. Why were Z scores used to assess differences between redeployed and non-redeployed staff on the variables of interest? Using a between groups t-test as a parametric test seems more appropriate here.

With regards to tables 3 and 4., it is unclear as to why each individual item has been assessed as a single variable. I recommend conducting a factor analysis/PCA to determine the number of individual factors that the 14 items assessed in table 3 and the 9 items assessed as per table 4 and then conduct the subsequent inferential analysis accordingly.

It is unclear why the F-stat p has been reported in tables 3 and 4. Given the type of analysis that has been reported, it would be beneficial to report the t-statistic here.

Overall, with regards to the results, I recommend providing more detail on the measures that were administered. Please ensure to conduct factor analyses/PCAs to demonstrate the factor structure of the measures used and which aspects of well-being were being assessed. This would help to conduct separate analyses based on the outcomes of the factor analyses/PCAs as opposed to using individual items to assess differences between deployed and redeployed staff. The discussion section will need to be revised as per the outcome of the factor analyses/PCAs and subsequent inferential data analysis.

VERSION 1 - AUTHOR RESPONSE

Reviewer 1

This study primarily relied on simple comparative statistics such as t-tests and Z-tests, which presents a clear limitation in that it did not adequately control for key confounding variables such as age, gender, tenure, job grade, and occupational group.

Future research would benefit from the following methodological enhancements: Strengthening control for confounding variables

RESPONSE: We most certainly recognise the scope for confounding variables, including, and not limited to, those identified by Reviewer 1. However, although substantial (and larger than any known alternative data sets) our sample is not of sufficient magnitude to support the suggested degree of interrogation.

We believe that the primary role of the article is to point to what presents as an indication of the need for deeper exploration of redeployment implications for NHS staff health, well-being and retention. We have added text (within the Human resources implications and Limitations sections) to emphasise this point. Relatedly we have suggested that the issue of redeployment might be incorporated as a variable in future waves of the NHS annual staff

survey; a data set of sufficient magnitude to support the exploration of potential confounding variables, including those identified by Reviewer 1.

Utilizing longitudinal data
Although the study collected data across four waves (2020–2023), the analysis was largely limited to cross-sectional comparisons. Leveraging the longitudinal design by applying repeated-measures ANOVA or mixed-effects models would allow researchers to explore temporal trends and interaction effects of redeployment over time, thereby strengthening both the explanatory power and persuasiveness of the findings.
Deepening causal exploration.

RESPONSE: The analysis focused on the most recent wave of data collection (Wave 4; Spring 2023). The justification advanced was of its greater contemporary relevance to the mature post-COVID-era return to routine care provision. Additionally, while the majority of questions were kept constant across the four waves ~10% were changed at each wave. This was due to the degree of flux and change arising from the pandemic and government policy changes to meet the information needs of key stakeholders (NHSE and DHSC) e.g. proposed mandatory vaccination of staff, fluctuations in infection rates, resumption of routine care.

To address this issue, additional explanatory text has been incorporated within the Materials and Analysis sections of revised draft.

Deepening causal exploration While definitive causal inference is not possible, structural equation modelling (SEM) or path analysis could be applied to examine indirect and mediating effects, such as “redeployment → increased stress → burnout → turnover intention.” Such an approach would provide richer interpretation beyond mean differences and help to clarify the pathways through which redeployment influences workforce outcomes, offering more practical insights for workforce management and policy interventions.

In sum, authors should be cautious not to overgeneralize the implications of the current analytical approach. Future studies should prioritize methodological advancements that strengthen confounder control, capitalize on the longitudinal nature of the data, and employ causal modelling strategies to deepen understanding of the mechanisms linking redeployment to staff outcomes.

RESPONSE: We agree there is potential for the above type of analysis to provide further illumination. However, we believe that this would be more appropriate for a subsequent bespoke study that set out to focus specifically on exploring impacts associated with redeployment, in particular the nature and frequency of redeployment across different segments of the NHS labour force. Our findings emerged as a component of the findings from a broader study of staff retention. We believe their contribution lies in pointing to the need for deeper consideration by policy makers, and investigation by research funders and researchers.

Text has been added to the revised draft to emphasise this point within the Human Resources Implications and Limitations sections.

Reviewer 2

In the summary of the method, could you please summarise the type of statistical analyses that were conducted to analyse the quantitative data. Add details

RESPONSE: Text detailing the types of statistic test applied have been added to the abstract and results section.

Strengths and Limitation - Please correct the spelling mistake of presentism, I assume that you mean presenteeism

RESPONSE: This spelling error has been corrected.

Note the misspelling ‘quantitive’.Fix typi

RESPONSE: This not a misspelling as both quantitive and quantitative are considered correct and synonymous by OED. However, we acknowledge the request and the term has been amended to quantitative.

Please check some of the typos and errors throughout.

RESPONSE: The revised draft has been proofread and typographical errors corrected where identified.

Introduction: Ensure to frame the introduction with a theoretical framework that provides an explanation as to why the process of being redeployed as healthcare professional may cause staff to consider leaving healthcare professions in the NHS.

RESPONSE: The ESRC grant proposal that initiated the study was cast as exploratory, grounded within the systems (job-demands, control support) risk mitigation perspective. As such it did not set out to test theory in a formal sense, but to explore widely cited variables associated with employee health, well-being with implications for staff retention within the context of prevailing working conditions and arrangements. The initial focus, in common with other commissioned research in response to the ESRC call for applied pandemic relevant studies, was on the exceptional COVID-19 pandemic demands placed on staff and the impact on their disposition to leave or stay. With the change in funders (NHS England Survey Waves 3 and 4, 2022/23) the focus evolved to legacy effects and the post-pandemic return to routine care delivery.

Our findings pointing to the relatively greater vulnerability of redeployed staff emerged as a notable feature of the survey findings, rather than being a variable for specific focus that we set out to test. The central finding is not that the redeployed and non-redeployed staff show divergent response profiles. Rather the patterns are essentially equivalent, but the salience / negative effect of variables is rated greater by redeployed staff, i.e. relatively higher stress, lower morale, stronger disposition to leave, etc..

Revised text has been added to the introduction and aims to reflect the above.

The term ‘functions’ in line 38/Introduction, should this not read as ‘services’ instead?

RESPONSE: We feel that the term ‘functions’ should be retained as it is a more generic and inclusive term than services.

With regards to the following “that are experiencing shortages due to absence or non-normal”, it would be useful to precede the word absence with ‘staff’ as it currently reads as though you are referring to an absence in the demand for care

RESPONSE: The word ‘staff’ has been added to this sentence.

Please support the following point with a relevant citation: “However, there are indications that the amplified rates of redeployment necessary during the COVID-19 pandemic persist and may become an increasingly encountered feature of working-life within the NHS”.

RESPONSE: The following references have been added to support this point:

RCN 2025 Redeployment and unsustainable pressures. https://www.rcn.org.uk/Get-Help/RCN-advice/redeployment-and-unsustainable-pressures

Department of Health and Social Care (DHSC) £7.5 million to digitally schedule shifts and save NHS staff time. Press release `5th January 2021. https://www.gov.uk/government/news/75-million-to-digitally-schedule-shifts-and-save-nhs-staff-time

The use of the word ‘sponsored’ appears to be a little odd in the following passage “COVID-19 working conditions sponsored notable redress”. Could you please rephrase accordingly. –

RESPONSE: The text been amended to ‘gave rise to’.

Please ensure to support the following point with a relevant citation: “COVID-19 working conditions sponsored notable redress in this respect, within the UK and internationally, typically as a component of broader perspectives on staff experiences, with a strong focus on the intuitively most challenging redeployments to emergency, intensive and end-of-life care.”

Response: the following citations have been added to the text: White et al., 2025 (ref 6); Hartley et al., 2024 (ref3); Dunning et al., 2025 (ref 7); Ballantyne and Achour, 2023 (ref 7); Kennedy et al., 2022 (ref 9).

Could you please ensure greater clarity in the following sentence: “While findings of positive staff experiences are also reported,4; 5 a negative profile dominates author accounts by volume and emphasis”.

RESPONSE: The text has been amended to: ‘Studies report an array of negative impacts on redeployed personnel arising, directly or indirectly, from pandemic working conditions and arrangements. While positive staff experiences are also reported, e.g. opportunity to enhance skills and experience[7; 9] negative impacts dominate study findings’

Ensure to clearly illustrate some of the potential benefits of being redeployed as indicated by the cited studies. The sentence above could also be reworded to ensure clarity.

RESPONSE: Addressed within response to preceding point.

Could you please write the following sentence more concisely: “The majority of studies focused on single health professions or functions and the impact of non-normal conditions during the height of the COVID-19 pandemic. They share the perspective of

characterising redeployed staff as a more vulnerable "…high-risk group that should be a target for health and well-being intervention" p.37812"

RESPONSE: This sentence has been reformulated to: 'The majority of studies focus on single health professions or functions and the impact of working conditions during the COVID-19 pandemic. They characterise staff redeployed during this period as a more vulnerable "…high-risk group so should be a target for health and well-being interventions" p.378[12].

The following sentence could also be more concise or broken down into a couple of sentences: "The profile of demand for care and prevailing conditions during the pandemic is reported to have sponsored a more centralised command and control structure, particularly within secondary care, with senior managers taking a more strategic role in staff deployment decisions, supported by accelerated rates of adoption of e-rostering technology, the latter reported to have "…proven instrumental when redeploying staff,... Specific skillsets can more easily be matched to the right shifts when redeploying staff …during the COVID-19 response to redeploy staff quickly to support areas in need."

RESPONSE: This paragraph has been amended as follows:

'The profile of demand for secondary care and prevailing conditions during the pandemic is reported to have sponsored a more centralised command and control structure, with senior managers taking a more strategic role in staff deployment decisions. An associated feature was increased adoption of e-rostering technology, reported to have "…proven instrumental,... during the COVID-19 response to redeploy staff quickly to support areas in need."[19] However, the post-pandemic persistence of this approach to staff deployment has been characterised as a cultural shift from historical norms that conflicts with staff expectations and devolved department-level decision making, e.g. by nurse managers[3].'

The introduction as a whole is mainly focused on the redeployment of healthcare staff during the COVID-19 pandemic as a means to cover service provision. There are a couple of sentences that illustrate that the redeployment strategy employed during the COVID-19 pandemic has persisted during the post-pandemic era. However, it would be useful to review more recent studies on staff well-being in healthcare professionals beyond the COVID-19 that are relevant to the variables that were assessed in the present study. As the present study also assessed the occupational well-being of non-deployed staff, it would be useful to review literature that illustrates how stability in workplace settings could be beneficial to the welfare of healthcare professionals in secondary care

RESPONSE: Weyman and O'Hara have checked for recent publications on the issue of redeployment and impacts on health and well-being with the NHS and more widely. Reiterating our conclusions within the paper, there appear to be few if any that relate to the late post-COVID / return to normal care delivery period, i.e. the focus of published findings remains limited to the pandemic period and COVID care redeployments. However, if Reviewer 2 is able to identify relevant publications we would be happy to consider acknowledging and integrating their findings.

Method - The following sentence could be more specific in stating intentions to stay or leave a position in the NHS as a healthcare professional: "Epistemologically, the research trajectory reflected a systems perspective, focused on contextual influences on employee health, well-being and disposition to stay or leave".

RESPONSE: We feel that this sentence should remain as originally configured. The terms systems perspective was used in our successful funding proposals to the (ESRC. and NHS England. The term systems perspective is widely used within the occupational risk literature on root causes and within the healthcare work and wellbeing literature to distinguish it from an individual perspective, e.g. Campbell Institute National Safety Council 2017 https://www.thecampbellinstitute.org/wp-content/uploads/2017/09/; Campbell-Institute-A-Systems-Approach-to-Worker-Health-Wellbeing.pdf; NHS Employer Evidence-based approaches to workforce wellbeing 2025 https://www.nhsemployers.org/articles/evidence-based-approaches-workforce-wellbeing and is present within the title of the UK Health and Wellbeing at Work Conference NEC Birmingham March 2026 'Taking a Systems Approach to Health and Wellbeing at Work: What Exactly Does That Mean?

The term disposition to leave was chosen as it more accurately reflects the data we have on staff behaviour as a precursor to leaving (e.g. submitted a job application), which is a more reliable predictor of behaviour than intention.

The following sentence could be reworded for clarity. Terms such as 'incubating precursors' read as rather convoluted here: "Taking a risk mitigation and control perspective, the focus was on identifying associative influences that can be characterised as incubating precursors with the potential to challenge staff resilience to prevailing working conditions/arrangements and diminish their attachment to NHS employment."

RESPONSE: We feel that this sentence should remain as originally configured. The term 'incubating precursors' has a specific accepted meaning that has been a in common usage within the organisations risk research domain since the 1970's. It is widely attributed to Barry Turner. - Turner, B. (1976) The Organizational and Interorganizational Development of Disasters, Administrative Science Quarterly, 21 378-397. Turner and Pidgeon Man-made Disasters Elsevier Science & Technology Books, 1997 London.

What is meant by "amplified vulnerability" in the following sentence? "The core objective was to identify priority issues for intervention, nuanced by identifying the demographics and functional groups of employees that exhibited amplified vulnerability." Could a simpler term, such as 'A core objective was to assess the demographics of healthcare professionals to identify if there are particular groups in the NHS who are vulnerable to leaving their profession':

RESPONSE: We feel that this the term 'amplified vulnerability' should remain as originally configured. There are a growing number of examples of it use within contemporary health risk publications e.g. Ferreia et al; Vulnerability and Primary Health Care: An Integrative Literature Review J Prim Care Community Health . 2021. Netrdova et al (2025) Exploring vulnerability amplification in regional health inequality: COVID-19 case study in Czechia (Applied Geography 177). The term amplified vulnerability was also chosen to point to the relatively greater vulnerability of redeployed staff – as such it acknowledges the valid point made by Reviewer 2 (above) that non-redeployed staff are also subject to a similar array of challenges to their health and well-being arising from prevailing working conditions and arrangements.

Ensure to not report results in the Method section as per the following sentence "Our initial headline analysis19; 20 revealed that redeployed staff exhibited a consistently more negative response profile on almost all issues covered by the survey".

RESPONSE: ”. After careful consideration we believe that the reference to ‘Out initial headline analysis…’ should remain within the method section as it relates to findings detailed within our previous publications on survey headline findings. Hence not in any specific sense arising from the analysis described within the current article – but was the basis of our rationale for exploring the issue of redeployment more deeply in the analysis reported here.

The paragraph has been reformulated to clarify this: ‘ Findings from our initial headline analysis of survey findings, reported elsewhere[21] indicated that redeployed staff exhibited a consistently more negative response profile across a high proportion of the variables explored in the survey. This provided the justification for the deeper exploration of the data reported in this paper.

Ensure to cite the studies/researchers that you are referring to here: “The relatively large sample allowed formal testing of associations and contrasts including those identified in (predominantly small sample; single occupation) studies by other researchers…”

RESPONSE: The following references have been added to the revised draft: Ballantyne and Achour 2023; (ref 8); Chan et al. 2005 (ref 26);J Nickell et al. 2004 (ref 27). Maunder R.. 2004 (ref28).

The sample
Does table 1 refer to data from healthcare professionals working in England only. i.e. was the data from UK services outside of England omitted from wave 1 and wave 2 for the analyses in this study?

RESPONSE: We conducted a comprehensive check on the response profiles between the; devolved nations. We found no differences in mean/ SD profiles (variations in response proportions ~1%). It was therefore considered appropriate to pool their data in any comparisons with the Waves 3 and 4 England only data, rather than unnecessarily diminish the available sample.

The following paragraph has been added to the revised draft: ‘The sample was stratified by occupational group and weighted by age, gender identity, ethnicity and region, with consistent representation by occupational group and type of care-provider organisation. The samples for Waves three and four were from England only. Waves one and two were UK-wide. Pre-analysis checks revealed no differences (proportions, means and standard deviations) in the response profiles between England and the devolved nations; indicating that the respective data sets could be treated as homogenous.’

The sample Ensure to include a Materials subsection that details the measures that were administered to staff over the four waves and how they were used to assess the variables of interest.

RESPONSE: A materials subsection has been added to the revised draft. A copy of the Wave four survey question set has also been added to the appendices, supplemented by an account of changes to the survey content (new, omitted and revised items) at each wave.

Analysis - The following section is a little unclear: “The findings focus primarily on the analysis from the fourth wave of data capture as the primary focus, due to their greater contemporary relevance”. Why was the focus on analysing cross sectional data at wave four –

RESPONSE: The focus on the Wave 4 data set was because the focus of the paper was on post-COVID pandemic working conditions. It is the most contemporary of the data sets, least subject to the abnormal demands of the pandemic, and therefore of greatest relevance

to policy makers and other sector stakeholders. Text has been added to the revised draft at a number points (identified elsewhere in the response to reviewers) to emphasise the rationale for focusing on the Wave 4 dataset.

Please ensure to be specific that the following change was applied at wave 4: The reference time frame was amended to ‘In the last 6 months, how often if at all have you been redeployed to a different location, department or team?’ (in place of ‘since March 2020’, used at the three earlier waves)
Could you also be more specific in this section and state the types of inferential statistical analyses that were applied in this study.

RESPONSE: The research was originally commissioned to track and monitor variables with the potential to impact on staff retention during the COVID-19 pandemic. The analysis reported in this paper uses the Wave 4 data set as the most contemporary data on working conditions and arrangements following the return to normal care delivery. Cross-wave comparisons have therefore been limited to:

(i) insight into the issue of whether pandemic redeployment rates (widely accepted as higher than the pre-pandemic norm) have reduced following the resumption of normal care delivery – which might be expected.
(ii) comparison of the ratio of voluntary to involuntary redeployments.
(iii) evidence of a marked decline in the proportion of staff who would recommend working for the NHS to others, which presents as a notable trend and we believe worthy of inclusion, with respect to both the redeployed and non-redeployed.

The section on analysis has been rewritten to address the above points as follows:

The analysis focuses on the fourth wave of data capture, (spring 2023) due to its greater contemporary relevance to working conditions and arrangements following the post-COVID-19 return to more-normal care delivery in late 2022/2023. At Wave 4, exposure to redeployment was determined on the basis of the response to the question ‘In the last 6 months, how often if at all have you been redeployed to a different location, department or team?’. The reference period at Waves 1, 2 and 3 was ‘since March 2020’. This change was introduced due to the increasingly distal anchor of the beginning of the pandemic, and the transition to more normal working conditions and arrangements.

In addition to descriptive statistics, inferential statistics tests (t-test independent samples, z test and Chi^2 trend test using SPSS 29.0-2.0[29]) were applied to compare redeployed with non-redeployed staff. Contrasts are explored for: rates of applications for non-NHS jobs and sickness presenteeism; ratings of stress, morale, ability to switch-off from work, symptoms of burnout; worry over current working conditions and confidence in improvement to working conditions.

Cross-wave comparisons are limited to the contemporary relevant issues of whether rates of redeployment (voluntary and involuntary) have reduced since the height of the pandemic in 2020/21 and the proportion of staff (redeployed and non-redeployed) who would recommend working for the NHS.’

Could the following sentence be stated with greater clarity: This change was considered necessary due to the increasingly distal anchor of the beginning of the pandemic, and transition to more normal working conditions and arrangements.

RESPONSE: This sentence has been amended as a component of the reformulated text detailed in the preceding response.

Results - Please ensure to state the analysis that was conducted to indicate that rates of involuntary redeployments had increased over the four waves as per the following: "At wave four, 24% of respondents said that they had been redeployed at some point in the previous six months. Of these, 25% reported this happening often and 75% occasionally. 30% of redeployments were reported to have been voluntary and 70% involuntary; with an indication of a rising rate of involuntary redeployments across the four waves" Ensure to report the inferential statistics in accordance with APA guidelines that clearly show how rates of involuntary redeployment increased over the four waves. As it is currently reported, it is unclear if the reported increased rates of involuntary redeployment were significant or non-significant.

RESPONSE: The results of a Chi^2 trend test have been added to this section of the results -

Ch^{i2} test for trend χ^2 (1, N = 794) = 15.45; p.0001).

Results With regards to the section on Figure 2. Why were Z scores used to assess differences between redeployed and non-redeployed staff on the variables of interest? Using a between groups t-test as a parametric test seems more appropriate here.

RESPONSE: Reference to Z 'Score' (page 8 para 1 I original draft) was in error. It should have read Z-test (to reflect how it was identified within each of the respective data tables within the reviewed draft). The Z test <u>is</u> a parametric test that is considered appropriate for use where proportions are approximately normally distributed and it is widely applied for large samples. Within the initial draft expressing findings as proportions was also judged to be a way of presenting findings in a more accessible and inclusive form for non-technical readers. However in acknowledgement of reviewer 2's request, within the revised draft contrasts are reported by reference to independent samples t-tests. As might be expected, the findings and interpretation remain substantively equivalent.

With regards to tables 3 and 4, it is unclear as to why each individual item has been assessed as a single variable. I recommend conducting a factor analysis/PCA to determine the number of individual factors that the 14 items assessed in table 3 and the 9 items assessed as per table 4 and then conduct the subsequent inferential analysis accordingly.

RESPONSE: The aim of the paper is to highlight the differences in response profile between the redeployed and the non-redeployed. We were not setting out to identify the primary constructs associated with the impact of redeployment on health and wellbeing. That would be more appropriately addressed via a dedicated study with a theoretically grounded question set focused on determining the primary impacts of redeployment.

The issue of redeployment was not the primary focus of our original study and was a contrast that emerged as a notable feature in our broader examination of staff retention. Consequently, our conclusion recommends the need for a more dedicated study of redeployment, that should take account of differential effects across different segments of the workforce and potential confounding effects of the type identified by Reviewer 1.

Additional text has been added to the redrafted abstract, HR policy implications and limitations sections of the revised draft to increase the emphasis on these points.

It is unclear why the F-stat p has been reported in tables 3 and 4. Given the type of analysis that has been reported, it would be beneficial to report the t-statistic here.

RESPONSE: t values (and means and standard deviations) have been added to tables 3 and 4.

Overall, with regards to the results, I recommend providing more detail on the measures that were administered. Please ensure to conduct factor analyses/PCAs to demonstrate the factor structure of the measures used and which aspects of well-being were being assessed. This would help to conduct separate analyses based on the outcomes of the factor analyses/PCAs as opposed to using individual items to assess differences between deployed and redeployed staff. The discussion section will need to be revised as per the outcome of the factor analyses/PCAs and subsequent inferential data analysis.

RESPONSE: We view a principal component analysis as beyond the scope of our dataset, with potential to generate partial and or misleading findings. It would be more appropriate for the variables required to support a PCA to be selected and tailored as part of a dedicated examination of redeployment. As we have detailed within the paper and the responses above – our data relates to a finding that emerged as a feature within our wider study of NHS staff well-being and is suggestive of the need for further large scale exploration of impacts on staff arising from redeployment. The intention of our paper is to highlight a research gap of potential importance for healthcare policy/practice in relation to staff wellbeing and retention.
